# Supplementary material for: Role of active patient involvement in undergraduate medical education: a systematic review
Source: BMJ Open. 2020 Jul 27;10(7):e037217. doi: 10.1136/bmjopen-2020-037217 (PMC7389514; doi:10.1136/bmjopen-2020-037217)
Supplement: Supplementary data [file bmjopen-2020-037217supp001.pdf]

Full search strategy

The authors performed a search through PubMed on July 12th 2018. The following search terms were used:

((“Patient Participation”[Mesh] OR "Community Participation"[Mesh] OR “Stakeholder Participation”[Mesh] OR “patient instructors”[tiab] OR “patient educators”[tiab] OR “patient teachers”[tiab] OR “patient-as-teacher”[tiab] OR patient participation [tiab] OR patient involvement [tiab] OR community participation [tiab] OR community involvement [tiab] OR patient engag [tiab] OR community engag* [tiab] OR patient cooperat* [tiab] OR community cooper* [tiab] OR patient collaborat* [tiab] OR community collaborat* [tiab] OR patient represent* [tiab] OR community represent* [tiab])*

AND

(“Education, Medical, Undergraduate”[Mesh] OR "Education, Medical"[Mesh] OR “Curriculum”[Mesh] OR “Students, Medical”[Mesh] OR medical education [tiab] OR medical student [tiab]))*

A limit was set to only show articles written in English and published between 2003 and 2018 to be included. Exclusion criteria were studies that did not address education to undergraduate medical students, where patients were only passively involved in education (for example, through presence at wards). Articles were first assessed based on title and abstract, and in a second round on full text. The references of all included articles were assessed for additional studies that matched the original inclusion criteria. All reviews that complied with the inclusion criteria were additionally assessed for relevant references, however, only original research articles were included.
