# Supplementary material for: Role of active patient involvement in undergraduate medical education: a systematic review
Source: BMJ Open. 2020 Jul 27;10(7):e037217. doi: 10.1136/bmjopen-2020-037217 (PMC7389514; doi:10.1136/bmjopen-2020-037217)
Supplement: Supplementary data [file bmjopen-2020-037217supp003.pdf]

Authors	Year of publication	Journal	Title	Country	Resarch question	Study design	Data collection	Setting	Outcome measure	# Patients in intervention	Patient characteristics	Role of patient organizations	Type of patient engagement	# Students in intervention
**Rees, C.E., Knight, L.V., Wilkinson, C.E.**	2006	Advances in Health Sciences Education	‘‘User Involvement Is a Sine Qua Non, Almost, in Medical Education’’: Learning with Rather than Just About Health and Social Care Service Users	UK	What are the views and experiences of stakeholders concerning user involvement in medical education?	Qualitative description	Focus groups	Medical school - general	Service user views and experiences on service user involvement in medical education	19	Health or social care service users	Medical educators explicitly preferred not to work with politicized groups of patients, but did include groups to hear multiple voices. Service users wanted to participate in groups in order to receive support.	Share views on service user involvement	
**Ivory, K. et al.**	2013	The Clinical Teacher	A clinical approach to population medicine	Australia	Not specified	Case study	Programme evaluation	Medical school - general	Description of the development of an integrated, community engaged population medicine curriculum		Patients with chronic health conditions	Partnership with consumer organizations to recruit patients	Contact with individual students during a six month period	
**Fritz, C. et al.**	2015	Journal of Racial and Ethnic Health Disparities	Are We Missing the Mark? The Implementation of Community Based Participatory Education in Cancer Disparities Curriculum Development	USA	What is the interest of community members in participating in curriculum design, how should they be involved in designing a cancer disparities curriculum, and what do community members believe the curriculum should address to positively impact their community?	Mixed methods - Exploratory design	Deliberative session and survey	Community forum	Stakeholder perspectives on creation of a cancer disparities curriculum for medical students and public health students	86	Members of Chicago community (focus on cancer)	Study concept directly derived from cancer support group	Curriculum development	
**Bideau, M et al.**	2006	Annals of the rheumatic diseases	Benefits of a programme taking advantage of patient-instructors to teach and assess musculoskeletal skills in medical students.	Switzerland	How effective is a rheumatid arthritis patient-instructor-based formation-assessment programme for its ability to improve and assess musculoskeletal knowledge and skills in third-year medical students?	Before and after study	Surveys and performance records	Patient-instructor programme to improve and assess musculoskeletal skills	Observed and reported student performance	11	All women, aged 29–66 years, who have had rheumatoid arthritis for at least 5 years.	Involved in patient recruitment	Active teacher, student assessor, feedback/coach	61
**Hatem, D.S., Gallagher, D., Frankel, R.**	2003	Teaching and Learning in Medicine	Challenges and Opportunities for Patients With HIV Who Educate Health Professionals	USA	What are the personal and programmatic effects of using HIV-infected persons as teachers in courses about care of HIV-infected people?	Qualitative - Phenomenological design	Interviews using semi-structured questionnaires	Course on HIV infection in which persons living with HIV cofacilitate small-group teaching and participate in panels	Patients responses on their motivation ad experiences	8	People living with HIV/AIDS, age 26-50		Curriculum development, development of learning material, active teacher	
**Barr, J. et al.**	2014	The Clinical Teacher	Committing to patient-centred education	Australia	Not specified	Case study	Programme evaluation	Programme where patient partners and students meet in weekly small group learning sessions facilitated by a clinician	Description of the program, implications, impact on learning, challenges		Patients from community practices (specialist and general)		Share experiences, give feedback on students' interviewing skills	
**Nestel, D. et al.**	2014	Advances in Medical Education and Practice	Community percpetions of a rural medical school: a pilot qualitative study	Australia	In what ways can [the medical school] engage with its local community? What are the community's expectations of [the medical school's] students hile completing their medical degree and upon graduaton?	Qualitative description	Interviews	Medical school - general	Expectations of lay community members	12	Any member of lay public, recruited through local newspaper advertisement		Interviewed to share perspective on community engagement	
**Owen, C., Reay, R.**	2004	BMC Medical Education	Consumers as tutors - legitimate teachers?	Australia	What is the feasibility of training mental health consumers as tutors for 4th year medical students in psychiatry?	Mixed methods - Convergent design	Programme evaluation	Programme where patients are trained as tutors and deliver inter a curriculum of interviewing skills	Patient, student and tutor feedback on their experience and attitudes	20	Consumers of mental health services	Partner in steering group and recruitment	Active patient teacher, curriculum development	104
**Kamaka, M.L.**	2010	Hawai'i Medical Journal	Designing a Cultural Competency Curriculum: Asking the Stakeholders	USA	What components should be included in a curriculum targeting Native Hawaiians?	Qualitative description	Focus groups	Medical school - general	Themes resulting from community focus grous	34 stakeholders (including students and physiicians)	Native Hawai'ians		Curriculum development	
**Baral, K.P., et al.**	2016	Journal of Nepal Health Research Council	Development of Community Based Learning and Education system within Undergraduate Medical Curriculum of Patan Academy of Health Sciences	Nepal	Not specified	Case study	Description of a curriculum development process	Medical school - general	A description and framework of curriculum development		Community members hosting students during field stay, local community leaders and local health workers involved in assessing students		Feedback/coach, student selection, teacher, assessor	

**Saketkoo, L. et al.**	2004	Teaching and Learning in Medicine	Effects of a disability awareness and skills training workshop on senior medical students as assessed with self ratings and performance on a standardized patient case	USA	What is the efficacy of a 3 hour workshop for medical students on disability skills and awareness?	Case-Control study	Surveys	A disability awareness and skills training workshop	Student self-efficacy ratings, knowledge of disability resources and medical complications, attitudes, confidence in ability to effectively advocate for patients with disabilities	3	1 team member with visual impairment, 1 attorney with mental illness, 1 parent of a nonverbal child	Partner in organizing team	Active teacher	147
**Noonan E.J. et al.**	2018	Teaching and learning in medicine.	Engaging the Transgender Community to Improve Medical Education and Prioritize Healthcare Initiatives	USA	What are common perceptions of transgender healthcare, priorities for improvement interventions, and themes to inform the curriculum?	Qualitative - Grounded theory design	Forum discussions with follow up questionnaire	Community forum	LGBT community views on transgender disparities in medical education	10-15	Transgender	Recruitment via local LGBT organizations	Curriculum development	
**Muir, D., Laxton, J.C.**	2012	Nurse Education Today	Experts by experience; the views of service user educators providing feedback on medical students' work based assessments	UK	What are the experiences of service users providing feedback to students on formative, workbased assessments?	Case study	Focus group with written feedback	Development and application of an assessment tool	Reflections by patient assessors following the analysis of student assessments using an interprofessional assessment tool	6	Lay people with experiences of ill health, caring and accessing health services	Lay group "The Patient Voice Group" of 6 members participated in the project	curriculum development, development of learning material, active teacher, student assessor, feedback/coach	253
**Hudson, G.L., Maar, M.**	2014	Rural and Remote Health	Faculty analysis of distributed medical education in Northern Canadian Aboriginal communities	Canada	Not specified	Case study	Programme evaluation	Mandatory Aboriginal community placement for all first-year medical students	Lessons learned and suggestions for curriculum improvement	7 communities	Aboriginal communities		Curriculum development, student assessor, feedback/coach, student selection	15
**Cheng, P., Towle, A.**	2017	Medical Teacher	How patient educators help students to learn: An exploratory study	Canada	What are the teaching practices and experiences that prepared patient educators for their roles in a longitudinal interprofessional Health Mentors program?	Qualitative - Exploratory design	Semi-structured interviews	Longitudinal interprofessional health mentors program	Experiences of and teaching methods used by interprofessional health mentors	11	Various conditions, 30-60 years		Active teacher	
**Gaver, A. et al.**	2005	Academic Medicine	Illness in context and families as teachers: A year-long project for medical students	Israel	Not specified	Case study	Student essays	Programme where second year students meet a volunteer family five times	Programme evaluation and student learning based on analysis of essays	100	Volunteer family, one of whose members suffers from a chronic disease or other impairment.	Involved in recruitment of families	Active teacher	40
**Bokken, L. et al.**	2010	Academic Medicine	Instructiveness of real patients and simulated patients in undergraduate medical education: a randomized experiment	Netherlands	Which contact (real patient or SP) is perceived as most instructive by students and which variables contribute to this?	Mixed Methods - Explanatory design	Quantitative questionnaires and qualitative focus groups	Volunteer sign up skillslab sessions with patient or SP encounters for first year students	Instructiveness to students (quantitative and qualitative)	9	Patients with asthma (age 54-82)		Active teacher, feedback/coach	163
**Cooper Director, H., Spencer-Daw, E.**	2006	Journal of Interprofessional Care	Involving service users in interprofessional education narrowing the gap between theory and practice	UK	Not specified	Case study	Students' reflective narratives, a focus group with practitioners, and individual semi-structured interviews with service users	Voluntary programme with four interprofessional student group workshops with e-learning and an introductory plenary.	Programme evaluation	35	Health or social care service users	Advise, provide assistance and support	Curriculum development, active teacher	~500
**Langlois, S., Lymer, E.**	2016	Education for Health	Learning professional ethics: Student experiences in a health mentor program	Canada	What are the experiences and learning of health profession students engaged in an ethics module as part of a health mentor program?	Qualitative - Phenomenological design	Student reflective papers and online discussions	Health mentor programme	Student experiences and learning	17	People with chronic health challenges		Active teacher, Feedback/coach	91
**Henriksen, A., Ringsted, C.**	2014	Advances in Health Sciences Education: Theory and Practice	Medical students' learning from patient-led teaching: experiential versus biomedical knowledge	Denmark	How do medical students perceive the experience of learning from ptient instructors in the context of coupled faculty-led and patient-led teaching sessions?	Qualitative - Grounded theory design	Focus group interviews	Medical school - general	Student perceptions	22	Patient Instructors associated with the Danish Association of Rheumatism	Employer of Patient Instructor leader	Curriculum development, development of learning material, active teacher	39

**Oswald, A.E. et al.**	2011 b	Medical Teacher	Musculoskeletal examination teaching by patients versus physicians: how are they different? Neither better nor worse, but complementary	Canada	How are musculoskeletal examination teaching by patients versus physicians different?	Case study	Video recording analysis	Medical school - general	Observed qualitative comparison of features and themes emerging from training	2	Lay people with a chronic MSK condition who undertake formal training in teaching musculo-skeletal physical examination skills		Active teacher	6 small groups
**Jackson, A., Blaxter, L., Lewando-Hundt, G.**	2003	Medical Education	Participating in medical education: views of patients and carers living in deprived communities	UK	What are the views of patients and carers living in deprived areas on their participation in medical education?	Qualitative - Phenomenological design	Semi-structured interviews	Patients' homes	Patient and carer views	54	Patients who live in socially and economically deprived areas, child carers, midlife patients and older adult patients		Active teacher	
**Coret, A. et al.**	2018	Teaching and Learning in Medicine	Patient Narratives as a Teaching Tool: A Pilot Study of First-Year Medical Students and Patient Educators Affected by Intellectual/Developmental Disabilities	Canada	What are trainees' experiences and learning outcomes resulting from exposure to video narratives followed by PE encounters?	Mixed methods - Convergent design	Focus groups, quantitative performance assessments	Video narratives by patients followed by patient-expert encounters	Qualitative and quantitative observed and self-reported data on student performance in clinical encounters	7	People living with intellectual and developmental disabilities (IDD)		Narrator	27
**Lucas, B, Pearson, D.**	2012	Education for Primary Care	Patient perceptions of their role in undergraduate medical education within a primary care teaching practice	UK	What are patients' perception of their role in undergraduate medical education within a UK primary care setting	Case study	Interviews	Primary care	Patients' perceptions	18	Patients who volunteered for involvement with pre-arranged undergraduate clinics		Active teacher	
**Oswald, A.E. et al.**	2014	Medical Education	Patient-centred education: what do students think?	Canada	What themes might be identified through a qualitative analysis of students' reflective writing on patient-centred education? What are common students' perceptions regarding patients as educators?	Qualitative - Grounded theory design	Students' written reflections	Patient Partners in Arthritis programme that teaches 4 hours of the 8 hours of pre-clinical MSK clinical skills small-group sessions	Student reflections on experiences of patient-educators		Volunteer patient educators with arthritis		Active teacher	356
**Towle, A., Godolphin, W.**	2013	Medical Teacher	Patients as educators: Interprofessional learning for patient-centred care	Canada	What issues are involved in creating an educational intervention designed and delivered by patients and document outcomes?	Case study	Focus groups with patient educators and staff, surveys with students	Interprofessional educational workshop focused on chronic disease management	Workshop design, student experiences and learning outcomes	24	Community Educators with epilepsy, arthritis, HIV/AIDS and mental health problems	Community organizations with a health mandate are part of Advisory Group	Curriculum development, development of learning material, active teacher	142
**Lauckner, H., Doucet, S., Wells, S.**	2012	Medical Education	Patients as educators: the challenges and benefits of sharing experiences with students	Canada	What are positive and negative factors that contribute to the experiences of patient-educators?	Qualitative description	Semi-structured focus group discussions and individual interviews	Mentor programme focused on patient -centred care, interprofessional collaboration, and chronic conditions and disabilities.	Conceptual framework of factors impacting patient experience	30	Adult volunteers with chronic conditions or disabilities. Mentors' conditions included diabetes, chronic pain, multiple sclerosis, cancer, stroke and various rare conditions.		Active teacher	
**Jain, S. et al.**	2013	Family Medicine	Patients with Disabilities as Teachers	USA	Not specified	Case study	Surveys with students	One hour session with patient with chronic disease facilitated by a faculty member	Description of programme	2	Persons with disabilities in their late 20's, who use wheelchairs; 1. spinal musclar atrophy, 1 cerebral palsy.		Active teacher	44
**Lazarus, P.A.**	2007	Medical Teacher	Patients' experiences and perceptions of medical student candidates sitting a finals examination	UK	What features of student candidates at finals do patients notice and consider when making judgements on them?	Qualitative description	Focus groups with patients	Finals examination of medical students before entering the clinical phase.	Patient perceptions and opinons of students during final examinatin	10	Patients, who had been involved in final examns, all >30 y.o, all white British ethnicity		Curriculum development and student assessor	
**Doucet, S., Lauckner, H., Wells, S.**	2013	Journal of Research in Interprofessional Practice and Education	Patients' Messages as Educators in an Interprofessional Health Education Program	Canada	What motivates patient educators to participate in a mentor programme and what messages they wanted to instill?	Qualitative description	Semi-structured focus groups	Mentor programme focused on patient -centred care, interprofessional collaboration, and chronic conditions and disabilities.	Thematic experiences of patient-educators	30	Community volunteers with a chronic condition and/or disability	Recruitment via patient networks	Active teacher	

**O'Keefe, M., Jones, A.**	2007	Medical Education	Promoting lay participation in medical school curriculum development: lay and faculty perceptions	Australia and NZ	What are lay and faculty perceptions around lay participation in medical curriculum development and how do they compare?	Qualitative description	Focus groups	Medical school - general	Identification of challenges of lay participation in medical curriculum development	17	Lay public		Curriculum development	
**de Boer, A. et al.**	2011	Clinical Rheumatology	Real patient learning integrated into a preclinical block musculosceletal disorders. Does it make a difference?	Netherlands	What are the educational effectiveness of the practicals, the expectations students have of real patients, and students' satisfaction?	Mixed model - convergent design	Formal written tests, surveys, focus groups	Non-obligatory practicals during a preclinical block on musculoskeletal disorders.	Educational effectiveness, student opinions and expectations, experienced effects	>4	Patients of the departments of rheumatology, orthopedic surgery, and/or patients under treatment of the local physical therapists.	Patients with spondylarthritis are members of a professional organization of arthritis educators which employs patients with RA in different phases of their disease.	Active teacher	377
**Baral, K. et al.**	2016	Cureus	Rural Community as Context and Teacher for Health Professions Education	Nepal	Not specified	Case study	Description of curriculum development	Describing an approach of ruralizing the academic medical education	Description of the program		Community members and leaders from rural areas, women's groups, CBOs, red cross, local health institutions		Curriculum development, selection of students, setting of school mission and vision, teaching	
**Gutteridge, R., Dobbins, K.**	2009	Nurse Education Today	Service user and carer involvement in learning and teaching: A faculty of health staff perspective	UK	What are staff views on current and ideal patient involvement?	Qualitative - Grounded theory design	Semi-structured interviews	Medical school - general	Academic and administrative staff views					
**Dogra, N. et al.**	2008	Medical Teacher	Service user perspectives about their roles in undergraduate medical training about mental health	UK	What are user perspectives on the specific role of service users in the delivery of teaching psychiatry?	Qualitative description	Focus groups	Medical school - general	Service user perspectives	28	16 men 12 women, all white adults, one of them a carer		Curriculum development	
**Jarvis-Selinger, S. et al.**	2008	Journal of Interprofessional Care	Social accountability in action: University-community collaboration in the development of an interprofessional Aboriginal health elective	Canada	Not specified	Case study	Description of curriculum development	Co-development of an Aboriginal Health course	Recommendations for curriculum development, issues and experiences	2 communities	Aboriginal leaders, instructors and Elders		Curriculum development	7
**McKinlay, E, McBain, L., Gray, B.**	2009	Chronic Illness	Teaching and learning about chronic conditions management for undergraduate medical students: utilizing the patient-as-teacher approach	NZ	What is the impact on medical student learning of a revised chronic conditions teaching programme?	Qualitative description	Surveys, reflective assays	Chronic conditions teaching programme based on the chronic care model utilizing	Student feedback on involvement of patients in teaching/home visit (before/after)		Patients with a specific, usually physical, chronic condition (such as diabetes)		Active teacher	57
**Humphrey-Murto, S. et al.**	2004	Teaching and Learning in Medicine	Teaching the Musculoskeletal Examination: Are Patient Educators as effective as Rheumatology Faculty?	Canada	What is the teaching effectiveness of patient educators compared to rheumatology faculty?	Randomized controlled clinical trial	A 9 station objective structured clinical examination.	The Patient Partner in Arthritis Program™ trained patient educators in a musculoskeletal examination course.	Musculoskeletal examination skills.	10	5 patient educators and 5 silent patients; patients with arthritis		Active teacher	62
**Towle, A. et al.**	2014	The Clinical Teacher	The expert patient as teacher: an interprofessional Health Mentors programme.	Canada	Not specified	Case study	Description of curriculum development	Interprofessional Education (IPE), Health Mentors Programme	Report on evaluation of ongoing programme		Patients with a wide range of conditions, including HIV/AIDS, arthritis, multiple sclerosis, spinal cord injury, cerebral palsy and mental health problems	Community organizations facilitate mentor recruitment	Active teacher, mentorship/coach	290
**Oswald, A.E. e.al.**	2011 a	BMC Medical Education	The impact of trained patient educators on musculoskeletal clinical skills attainment in pre-clerkship medical students	Canada	Are there differences in physical examination skills between physician and patient taught students?	Randomized controlled clinical trial	Objective Structured Clinical Examination and questionnaires.	The Patient Partner in Arthritis Program™ trained patient educators in a musculoskeletal examination course.	Musculoskeletal examination skills.		Volunteer patient educators with arthritis		Active teacher	83

**Jaworsky, D. et al.**	2017	AIDS Care	The role of people living with HIV as patient instructors – reducing stigma and improving interest around HIV care among medical students	Canada	What is the impact of patient-facilitated education on reducing HIV-related stigma?	Before and after study	Health Care Provider HIV/AIDS Stigma Scale (HPASS) questionnaire	Simulated clinical encounter in which medical students provided HIV pre- and post-test counselling.	Stigma on HIV and comfort in providing HIV care	16	People living with HIV/AIDS	PHAs were recruited through a community partner, the Toronto People With AIDS Foundation	Active teacher	67
**Haq, I. Fuller, J., Dacre, J.**	2006	Rheumatology	The use of patient partners with back pain to teach undergraduate medical students	UK	What is the impact of teaching about back pain to medical students using trained patient partners?	Randomized controlled clinical trial	Objective Structured Clinical Examination and questionnaires.	Teaching about back pain to medical students by trained patient partners, during at 5-week rheumatology/orthopaedics firm.	Musculoskeletal examination skills.	4	Three male patients and one female , age 29–56. Ankylosing spondylitis and chronic back pain with intermittent sciatica.		Active teacher	60
**Collins, L. et al.**	2011	Journal of Interprofessional Care	Transforming chronic illness care education: A longitudinal interprofessional mentorship curriculum	USA	What are student reflections on the impact of a longitudinal mentor program with a chronic condition?	Qualitative description	Students reflection assays.	Interprofessional Health Mentors Program and Chronic Care Model	Student perspectives on the programme and chronic illness		Patients with chronic illness or disability		Coach/mentorship	60
**Raj, N. et al.**	2006	Rheumatology	Undergraduate musculoskeletal examination teaching by trained patient educators—a comparison with doctor-led teaching	UK	How do hand and knee examination skills gained by undergraduates taught either by trained patient educators and physicians compare?	Randomized controlled clinical trial	Objective Structured Clinical Examination and questionnaires.	Students were taught an established core skills set in small group workshops. Students	Musculoskeletal examination skills.	12	11 female, 1 male, age 34–79. Rheumatoid arthritis, osteoarthritis, inflammatory arthritis.		Active teacher	50
**Tierney, W.M.**	2018	Journal of General Internal Medicine	Use of Stakeholder Focus Groups to Define the Mission and Scope of a new Department of Population Health.	USA	Not specified	Case study	Description of curriculum development	Input for strategic development of department of population health in new medical school	Consensus on focus areas for the school's curriculum	13	Local community non-profit organizations	People recruited from local community organizations	Curriculum development	
**Duggan, A. et al.**	2009	Journal of Health Communication	What can I learn from this interaction? A qualitative analysis of medical student self-reflection and learning in a standardized patient exercise about disability	USA	How does reflective practice about patients with disability promote relationship-centered care?	Qualitative - Grounded theory design	Videotaped feedback sessions between student en simulated patient with disability	Simulated patient encounter with a patient with an apparent disability.	Student reflections and insights gained	12	Patients with an apperant disability; included Multiple Sclerosis, Juvinile rheumathoid arthritis, traumatic spinal cord injury, neurodegenerative disorders, cerebral palsy, muscular dystrophy, blindness, paraplegia, quadriparesis, respiratory failure, NF and mobility constraint		Standardized Patient Educator	138
**Jha, V. et al.**	2009	Medical Education	What educators and students really think about using patients as teachers in medical education: a qualitative study	UK	What are the views and experiences of medical tutors and students of involving patients?	Qualitative description	Semi-structured interviews	Medical school - general	Student and medical tutor views and experiences on patients as teachers in medical training				Curriculum development, active teacher, assessor	28
**Alahlafi, A., Burge, S.**	2005	British Medical Journal	What should undergraduate medical students know about psoriasis? Involving patients in curriculum development: modified Delphi technique	UK	Not specified	Case study	Literature review and modified Delphi technique.	Medical school - general	Consensus among patients/physicians/nurses on content of the psoriasis workshop.	20	Patients with psoriasis		Curriculum development	
**Stagg, P., Rosenthal, D.R.**	2012	Rural and Remote Health	Why community members want to participate in the selection of students into medical school	Australia	What motivates community members to participate in the selection of medical students?	Qualitative description	Semi-structured interviews	Community member participation in selection of medical students	Patient experience and perceptions	8	Community Liaison Committee members		Student selection	
