# Supplementary material for: Role of active patient involvement in undergraduate medical education: a systematic review
Source: BMJ Open. 2020 Jul 27;10(7):e037217. doi: 10.1136/bmjopen-2020-037217 (PMC7389514; doi:10.1136/bmjopen-2020-037217)
Supplement: Author's manuscript [file bmjopen-2020-037217.draft_revisions.pdf]

BMJ Open is committed to open peer review. As part of this commitment we make the peer review history of every article we publish publicly available.

When an article is published we post the peer reviewers' comments and the authors' responses online. We also post the versions of the paper that were used during peer review. These are the versions that the peer review comments apply to.

The versions of the paper that follow are the versions that were submitted during the peer review process. They are not the versions of record or the final published versions. They should not be cited or distributed as the published version of this manuscript.

BMJ Open is an open access journal and the full, final, typeset and author-corrected version of record of the manuscript is available on our site with no access controls, subscription charges or pay-per-view fees (http://bmjopen.bmj.com).

If you have any questions on BMJ Open's open peer review process please email info.bmjopen@bmj.com

BMJ Open

Active patient involvement in undergraduate medical education: A systematic review

Journal:	*BMJ Open*
Manuscript ID	bmjopen-2020-037217
Article Type:	Original research
Date Submitted by the Author:	02-Feb-2020
Complete List of Authors:	Dijk, Stijntje; Erasmus MC<br>Duijzer, Edwin; University Medical Centre Groningen<br>Wienold, Matthias; Wissenschaftliche Dienste und Projectbaratung
Keywords:	EDUCATION & TRAINING (see Medical Education & Training), MEDICAL EDUCATION & TRAINING, HEALTH SERVICES ADMINISTRATION & MANAGEMENT

SCHOLARONE™
Manuscripts

I, the Submitting Author has the right to grant and does grant on behalf of all authors of the Work (as defined in the below author licence), an exclusive licence and/or a non-exclusive licence for contributions from authors who are: i) UK Crown employees; ii) where BMJ has agreed a CC-BY licence shall apply, and/or iii) in accordance with the terms applicable for US Federal Government officers or employees acting as part of their official duties; on a worldwide, perpetual, irrevocable, royalty-free basis to BMJ Publishing Group Ltd ("BMJ") its licensees and where the relevant Journal is co-owned by BMJ to the co-owners of the Journal, to publish the Work in this journal and any other BMJ products and to exploit all rights, as set out in our licence.

The Submitting Author accepts and understands that any supply made under these terms is made by BMJ to the Submitting Author unless you are acting as an employee on behalf of your employer or a postgraduate student of an affiliated institution which is paying any applicable article publishing charge ("APC") for Open Access articles. Where the Submitting Author wishes to make the Work available on an Open Access basis (and intends to pay the relevant APC), the terms of reuse of such Open Access shall be governed by a Creative Commons licence – details of these licences and which Creative Commons licence will apply to this Work are set out in our licence referred to above.

Other than as permitted in any relevant BMJ Author's Self Archiving Policies, I confirm this Work has not been accepted for publication elsewhere, is not being considered for publication elsewhere and does not duplicate material already published. I confirm all authors consent to publication of this Work and authorise the granting of this licence.

For peer review only

Title: Active patient involvement in undergraduate medical education: A systematic review

Stijntje W. Dijk[1], Edwin Duijzer[2], Matthias Wienold[3]

[1] Medical student, Erasmus University Medical Center, Doctor Molewaterplein 40, 3015 GD Rotterdam

[2] Medical doctor, University Medical Center Groningen, Hanzeplein 1, 9713 GZ Groningen

[3] Patient advocate, Wissenschaftliche Dienste, Rigaer Straße 30, 10247 Berlin

Corresponding author:

Stijntje W. Dijk, Erasmus University Medical Center, Doctor Molewaterplein 40, 3015 GD Rotterdam

email address: 340974sd@student.eur.nl

For peer review only

Abstract

OBJECTIVES: *To identify the scope of active patient involvement in medical education, addressing the current knowledge gaps relating to short and long term learning outcomes, ethical issues, psychological impact and key procedural contributors like recruitment, selection and preparation.*

DESIGN*: A narrative systematic review of publications between 2003 and 2018 in which patients take on active roles in the development, delivery or evaluation of undergraduate medical education.*

RESULTS*: 49 articles were included in the review. The new body of empirical evidence shows the wide range of learning objectives were pursued through patient participation, including competencies as professional, communicator, collaborator, leader and health advocate, but not scholar. Patients were engaged in a variety of educational settings in and outside of the hospital. The vast majority of studies describe patients taking on the role of a patient-teacher and formative assessor. More recent studies suggest that patients are increasingly involved in course and curriculum development, student selection and summative assessment. Measures to support sustainable patient involvement included longitudinal institutional incorporation, patient recruitment and/or training, resource support and clear commitment by faculty. The importance and advantages of patient involvement were highlighted by students, faculty and patients themselves, however, organizations must continue to consider, monitor and take steps to mitigate any potential harms to patients and students.*

CONCLUSION*: This systematic review provides new knowledge and practical insights to physicians and faculty on how to incorporate active patient involvement in their institutions and daily practice, and provides suggested action points to patient organizations wishing to engage in medical education.*

Article Summary

Strengths and limitations

- This systematic review is the first of its kind focused specifically on undergraduate medical education, providing practical guidance to educators, students and patients with ambition to improve work in healthcare professionals education.
- The study provides novel insights in the wide range of learning objectives pursued through patient participation, the educational settings and roles in which patients participate, and practical support systems that enable patient engagement.
- The study is limited to peer reviewed published literature in scientific journals. As many articles written by patients on their experiences in involvement in medical education may only be found in grey literature, including blog posts, conference statements and patient organization newsletters, this review may have missed their viewpoints.

Introduction

In recent decades, the involvement of patients in medical education has been advocated for increasingly and has become common practice adopted by reformers of medical education. (1) Patients and their narratives are no longer just used as subjects for "learning material" in clinical training. Towle et al. identified different levels of involvement, from paper based involvement to involvement at the institutional level as co designers of the medical curriculum in addition to sustained involvement as patient-teachers in education, evaluation and curriculum development. (1-4) Medical educators are now seeing the value of linking medical students with patients and their families and communities to foster awareness of the importance of longitudinal relationships, to improve students social interaction skills and to facilitate learning of coping with illness in the real world. (5, 6)

Despite an increasingly collaborative role of patients in medical education, there is much to be learnt about how to embed it, and how to develop systematic, institution-wide approaches to planning patient involvement in all levels of medical education. (3, 7) The drive towards a more equal partnership in clinical decision making and patient-centered care, fueled by national and international guidelines, promote the expansion of the efforts towards developing a culture where partnership in medical education becomes the norm. (3, 5-15)

As researchers have used varying definitions of active patient involvement in medical education, they have used varying inclusion criteria in their literature searches. This has resulted in overlap of included papers, and limited the generation of a common theoretical framework and terminology. (3, 16) Previous studies have identified major gaps in the knowledge base relating to short and long term learning outcomes, ethical issues, psychological impact and key procedural contributors like recruitment, selection and preparation. There is also limited information of the cost-effectiveness of active patient involvement.

Since the publication of the last systematic reviews (1, 17, 18) and non-systematic reviews (2, 8, 19) of patient involvement in medical education many, new studies have been published. Previous reviews addressed only the patient teacher role (1), teaching and assessing one specific skill (intimate examination) (17), included simulated patients (17), included post-graduate medical education (18) or addressed all health care professions. (2, 17, 19) Our paper reviews and summarizes the most recent literature using a broad definition of patient involvement consisting of any form of involvement that is beyond merely incidental passive involvement, in any field or setting of undergraduate medical education. By adopting this definition we have been able to extend the scope and amount of research data in order to increase the practical knowledge base on active patient involvement and in order to give ground to an improved theoretical framework and common terminology.

Methods

Design

Our literature search employed a systematic review method looking for active patient involvement in medical education defined as the direct, involvement of real patients and community members in the development, delivery or evaluation of undergraduate education of medical students.

Search Strategy

The authors performed a search through PubMed on July 12th 2018. The search terms used on their own and in combination included*: patient*, communit*, involvement, engag*, cooperat*, collaborat*, represent*, medical education, curriculum, medical student*.* Search criteria were reviewed by a hospital information specialist. The full search strategy can be found in **[SUPPLEMENTARY FILE 1]**

All articles published in English between January 1st 2003 and July 12th 2018 and reporting primary empirical research that addressed the active participation of patients in undergraduate medical education were eligible for inclusion. Studies with simulated patients or actors, patients solely undergoing examinations or patients who were only being observed in wards were excluded from the review. We assessed articles based on title and abstract in the first round, and in a second round based on full text. References of all included articles were analyzed for additional studies that matched the original inclusion criteria. All reviews that complied with the inclusion criteria were additionally assessed for relevant references. Only original research articles were included in the final analysis.

Results

The initial search resulted in 769 articles, of which 95 were selected for further review based on the title and/or abstract. These 95 articles were independently read by two authors (SD and ED) and included based on the specified criteria. Consensus between

For peer review only - http://bmjopen.bmj.com/site/about/guidelines.xhtml

reviewers was 91,2%. The remaining articles were included based on consensus after a short discussion. Main reasons for exclusion where wrong article type and studies that did not concern active participation of patients. The review of references resulted in 11 additional articles for inclusion. A total of 49 articles were included in the review. [**FIGURE 1, SUPPLEMENTARY FILE 2**]

Rationale for involving patients in medical education

Several authors referred to government policy mandating patient participation in medical education, namely the UK Department of Health and the UK General Medical Council (16, 20, 21), the Australian Medical Council (22) and the World Health Organization (WHO) (5) as a rationale for their patient involvement initiatives. Besides these political drivers, cited rationales were: to teach students patient-centered and interprofessional care (21, 23-25); to introduce students to chronic illness care (26-28); to create a multicultural learning environment; to practice social accountability and an inclusion agenda (29-34); to make education more engaging, powerful, and transformative (35); and to empower patients. (36) Patients mentioned that they felt a sense of responsibility to the broader community in shaping the future health workforce (21, 29) and improving the healthcare system. (21, 30, 37)

Patients recruitment and selection for involvement in education

The most frequently reported methods to recruit patients were through existing university partnerships and existing programmes (25, 30, 38-43), advertisements through press or social media or posted in health facilities (23, 31-33, 44, 45), through community- and patient organizations, through personal connections and previous participants (29, 35, 46-48), and through health professionals (49-52). In one project, where students shadowed a patient with a chronic condition, students were asked to recruit patients themselves. (27)

Selection criteria were generally set up broadly, inviting any patient or community member. General criteria for patient educators included good communication skills, affinity for teaching, aptitude for further learning, enthusiasm, time to commit to the study, as well as being fully mobile and being able to cope with repeated physical examinations. (45, 51, 53) In some cases patients were required to have representative physical signs of their disease. (50, 51, 53, 54)

Patients preparation for their roles in education

20 articles mention preparation of patient teachers. The duration of the preparation ranged from substantial training sessions of 100 hours in total (39) to the majority of programmes providing solely written information or a single orientation session of 1 to 1.5 hours (21, 27, 29, 36, 40, 46, 49, 54). Training programmes for patients in teaching musculoskeletal skills were the most extensive and were delivered by medical or educational experts. (39, 44, 51, 53, 55, 56) Other preparatory sessions were less formal and were facilitated by faculty educational experts, students or peers.

The primary aims of the preparatory programmes varied. One study underscored the importance of patient educators being aware of the course goals in order to safeguard student learning outcomes. (50) Others mentioned aiming to serve the needs of patients in building their confidence, providing skills training (54) and providing knowledge related to the educational process. (20, 36, 45, 57) These sessions addressed approaches such as problem based learning, how to deliver a presentation, co-facilitation methods, and how to provide effective feedback. Preparation also provided opportunities for anticipating benefits and challenges such as conflicts, emotions, unmet expectations, using methods of coaching, supervision and debriefing. (37, 58)

One article mentions finishing the preparation of patient educators with a short quiz as an assessment tool and having a graduation session before starting to teach. (25) Another article describes the use of a post-training satisfaction questionnaire to help ensure that patient educators felt ready to teach. (44) The majority of papers did not address assessing patients prior to them taking on their roles.

Towle et al. (2013) discussed the tension between preparedness of patient educators and authenticity of education in both form and content. (46) They highlight the critical role of the community organization representatives who can be brokers between the two cultures of academia and community. One article describes an intervention in which the patient educators explicitly have not received training, so the student-patient encounters would be as authentic as possible. (50) Another article described that the collaboration between patients and educators allow for mutual learning without an authentic patient perspective being lost. (36)

For peer review only - http://bmjopen.bmj.com/site/about/guidelines.xhtml

What are the roles in which patients can be involved in medical education?

The main categories of roles that we identified are divided in the areas of a patient as a teacher, an assessor, a curriculum developer and a student selector. [**BOX 1**]

Key patient roles in medical education

1. Patient as teacher

The role of a teacher was cited most frequently. Patient teachers gave clinical skills practicals on history taking and physical examination sessions on their own condition such as musculoskeletal disorders. (39, 44, 51, 53, 55, 56, 59) They were trained to teach students skills and deliver immediate feedback, which stimulated further learning. (53)

Several groups of patients with disabilities acting as patient teachers with disabilities gave practicals on communication skills and history taking. (45, 50, 54) People living with HIV participated as teachers during a simulated clinical encounter in which students provided counseling. (57)

In addition to clinical skills, patients taught students about their experiences of overall management of care, and the personal aspects of their lives. These ranged between practical physical and home adjustments, to psychological, social and behavioural issues impacting them and their family. Teaching was done through panel discussions and small group sessions (21, 40, 60) as well as visits to the community and patients' homes. (28, 49) Patient teachers with chronic conditions acted as mentors, and met regularly with students. (24, 26) Patients taught students patient-centeredness and interprofessionalism (20, 21, 28, 35, 46), community-centeredness, cultural competence and ethics. (30, 61) Patients could choose their own teaching method, such as telling their stories and stimulating reflection. (35) A group of patients living with intellectual and developmental disabilities also participated in the creation of learning materials, through videos sharing their perspectives and stories. (62)

Most patients in the study by Jackson et al. (2003) considered themselves not as teaching, but having a role of partnership, explanation and sharing certain aspects of their illness. (49)

2. Patient as assessor of students' competence

In addition to formative student assessment, such as feedback during teaching sessions, patients participated in high stakes summative assessments, such as the final year Objective Structured Clinical Examination (OSCE). (36) Patients also provided written feedback to student essays, which were used for formal assessment. (36) Patients assessed mostly non-cognitive domains of student performance. (32, 43) Medical educators interviewed by Jha et al. (2009) believed there was a role for patients in assessing whether students made them feel at ease and whether students asked the right questions. (63) Whilst patients and medical educators in the study by Raj et al (2006) praised patients' assessments, students expressed their concerns whether patients could reliably assess clinical skills or whether they were likely to be too lenient. (51)

3. Patient role in curriculum development

Community members were motivated to participate in curriculum development. (32) While they were not seen as medical experts, they did have an interest in ensuring optimal health care for themselves and their families. (23)

Community members actively participated in the planning, implementation, and evaluation of the educational program. (43, 46) Patient-teachers had autonomy from the stage of planning to the stage of delivery of teaching. (40, 64) Several patients were members of the steering committee for the psychiatry curriculum (25) and the interprofessional education curriculum. (46)

Patients were involved in the development of courses related to their illness or social conditions. (32) Aboriginal delegates provided recommendations for the development of an Aboriginal health curriculum and community placement. (30, 41) Focus group meetings with Native Hawaiians were held to define a cultural competencies and health disparities curriculum. (34) A world café discussion was similarly held to inform the curriculum on transgender health. (33) Minority community members provided input on curricular design, especially on the content of the cancer disparities curriculum. (32)

Beyond the disease- or competency specific courses, patients were involved in consultative meetings with stakeholders to identify desirable attributes, competencies of graduates, and development of a community based learning environment. (42) Patients were

For peer review only - http://bmjopen.bmj.com/site/about/guidelines.xhtml

For peer review only

also consulted on the desired characteristics of the curriculum. (65) One medical school sought input for the strategic development of the department of population health in a new medical school through focus groups. (66)

4. Patient role in selection of students to medical schools

Community members were invited to join a panel together with clinicians and academic staff members to select students applying for the Graduate Entry Medical Program. (29) Members of the community were invited to be a part of the student selection process and team, especially in assessing candidates' communication skills as well as sensitivity, compassion and empathy towards social contexts and societal needs. (42)

Collaboration between faculty and patients

The role of faculty members in the collaboration with patient teachers varied. Some patient teachers worked under the supervision of a clinical preceptor. (57) In other sessions, patients were co-facilitators with practitioners. (20) Workshops were led by patient teachers and facilitated, but not controlled by faculty. The faculty member's role was to support the direct learning between students and mentors. (46) Faculty was not always present in meetings but could provide background support, such as setting broad topics for discussions. (43) Patient-teachers stated that program support was essential for participation, allowing them to transform from teaching individual messages to teaching universal lessons. (40)

Lay participants of one study regarded sharing of curriculum ownership as necessary to acknowledge the importance of lay perspectives, whereas faculty participants presumed ownership of curriculum development. (23) Faculty in the study by Jha et al. were not clear on how to involve patients more fully in assessments or course development, nor were they convinced of the appropriateness of doing so. Some faculty members expressed their experiences of working with patient-assessors and course developers as tokenistic. (63)

Learning objectives achieved through active patient involvement

Learning outcomes of patient participation were quantitatively assessed on the subject of musculoskeletal (MSK) examination skills in four randomized controlled experiments (39, 44, 51, 59) and two further studies. (53, 56)

No difference was observed in increased structured clinical examination (OSCE) scores when comparing sessions delivered by trained patient educators with sessions delivered by rheumatology staff together with a passive patient undergoing examination (51, 59) and sessions with a non-MSK specialist physician (39).

In the experiment by Humphrey et al., significantly fewer faculty-taught students failed (0 out of 32) than patient educator-taught students (5 out of 30). (59) Students rated faculty educators higher than patient educators (4.13 versus 3.58 on a 5-point Likert scale). (59)

When students were taught by a patient teacher in addition to the regular faculty-led sessions, their OSCE scores increased more compared with students participating in the regular curriculum. (44) An intervention by de Boer et al. (2011) offered students the opportunity to participate in two non-obligatory real patient learning sessions in the preclinical musculoskeletal disorders block. (56) Students who participated scored significantly higher at the end-of-block test.

Oswald et al. (2011) examined how teaching was different between patient educators and physician educators when teaching MSK physical examination skills. (55) Video recordings show that trained patient educators were more consistent in content and style by consistently covering all major joints. Bokken et al. (2010) assessed student's perspectives on instructiveness of real patients versus simulated patients. (50) Overall instructiveness was marked high. Students regarded real patients as more authentic and the encounters more useful in practicing physical examination

In the intervention study by Jaworksy et al (2017) medical students provided HIV test counseling to patient instructors. (57) Pre- and post-intervention scores of the validated Health Care Provider HIV/AIDS Stigma Scale (67) demonstrated a significant decrease (68.74 versus 61.81). Students reported increased comfort in providing HIV-related care (10.24 versus 18.06). Similarly, students in intervention studies with patient teachers living with physical or mental disabilities, demonstrated an improved attitude (25), increased levels of comfort in communication (54), increased levels of self-efficacy and confidence (60, 62) and higher mean performance scores across all interview stations when compared to a control group. (62)

Students in the study by Rees et al. described the encounters with patients as more motivating compared to textbook learning. (68)

For peer review only - http://bmjopen.bmj.com/site/about/guidelines.xhtml

Wide ranges of learning outcomes of education with patient participation were mentioned in the qualitative studies identified by this review. To explore this range of outcomes a categorization is used according to the CanMEDS™ framework, developed by the Royal College of Physicians and Surgeons of Canada. (69) [**BOX 2**]

Communicator

Several authors mentioned patient-centered care as the main outcome of education involving patients. (20, 24, 26, 46, 64) Patient-centeredness included the ability to see patient-mentors as individuals (24), the importance of patient autonomy and expertise in care (61), adopting a non-patronizing and nonjudgmental attitude (52), recognizing patients' needs (38) and seeing the patient as a capable part of the team. (38) Jha et al. pointed out that active patient involvement by itself demonstrates an equal partnership (63) and Rees et al. concluded that this approach helps students to develop a holistic perspective of health care. (68)

More generally, patient participation was associated with increased understanding of the importance of communication (24, 26), building and improving communication skills (52, 68), empathy, listening skills and respect. (68)

Collaborator

McKinley described an education programme in which students undertake a home visit to a patient with a chronic condition (28), where students demonstrated increased understanding of interdisciplinary teams in management of chronic conditions in their reflective assays. Four authors described interprofessional education programmes in which patients are involved. (20, 24, 41, 46)

Leader

In a longitudinal mentor program with medical, physical therapy, occupational therapy, nursing, and pharmacy students teaming up with a patient-mentor students reported a deeper understanding of the healthcare system. (24). A yearlong student mentor program gave students an experience in and appreciation of continuity of care. (52)

Professional

Various qualitative studies suggested that patient involvement can attribute to dealing with ethical complexity in clinical practice and patients' perspectives on clinician error (61) and developing reflective skills. (26, 45, 52) Reflecting on role models some authors referred to broadening understanding of the role of the health care provider (24), qualities of remarkable clinicians that inform personal ideals (61), creating a future professional model (52) and professional identity. (68) Experiences with real patient-educator encounters could also help in coping with uncertainties, emotions and stress. (68)

Exposure to patient-educators from within specific patient or minority groups helped students to increase positive attitude toward chronic conditions and elderly (24, 28), patients with mental health problems (26) or disabilities. (45)

Health advocate

Students reflected on the importance of patient advocacy in day-to-day practice in a study on experiences within an ethics and professionalism module with patient-mentors. (61) More specifically, students were empowered to advocate for patients when they are in vulnerable situations. One of the aims of the education program described by Saketkoo et al. was to develop an awareness of the impact of physician advocacy, specifically in the context of people with disabilities. (60) A pre- and post-test showed that this awareness increased significantly with the programme.

Scholar

No programmes have explicitly described the aim of developing the competency of scholar.

Medical expert

The role of medical expert integrates all other roles by applying medical knowledge, clinical skills, and professional values in the provision of high-quality and safe patient-centered care. Two articles mentioned that patient participation supports students' learning, by recognizing the complexity of practicing medicine. (38, 45) Jha et al described the patient as providing an illustration of the theory in practice, thus enhancing students' understanding and recall. (63)

For peer review only - http://bmjopen.bmj.com/site/about/guidelines.xhtml

Concerns about the involvement of patients

Various authors have also raised concerns when involving patients as teachers in medical education. Some faculty educators were concerned that patient stories might be so traumatic that students would require support or debriefings to deal with the resulting emotions. (63, 68) . In a qualitative study, students felt "pressured" by service users asking them for information and advice, rather than asking their clinicians, or when service users divulged information to students that they had not told their clinicians. (68) Students worried about giving incorrect information to patients.

Students expressed reservations that they were only getting the view of one person, which could lead to a biased perspective. (20) Students were also concerned that patients might have difficulty discriminating between poor and good performance, and are likely to be too lenient in feedback or assessment. (51) Students in the study by Henriksen and Ringsted (2014) expressed skepticism about patients' knowledge (64) and expressed concerns about unstructured experiential learning in a context where patients had autonomy in both planning and delivering the teaching encounter. (64) In a different study, staff members expressed the concern that the impact of the patient experience might be reduced if the same patient was involved in the same programme too often. (63)

For peer review only

Patients' views of the impact of their involvement

Patients described a strong sense of having a meaningful contribution and personal fulfillment, because they were teaching patient centeredness (21, 70), offering their body and authenticity, bolstering students' confidence, (70) fulfilling their responsibility to the broader community (21, 29) and improving the health care system. (21, 30, 37)

On an individual level patients described material, professional, personal and emotional benefits. Material benefits included receiving tangible rewards such as gifts (40) and receiving a full medical checkup. (37) Patient educators with back pain involved in teaching medical students stated their participation improved the management of their own back pain, and improved confidence in voicing their needs in consultations with physicians. (44) Some patients felt that they received more time and attention from their healthcare professionals when they were teaching. (68)

Patients described professional growth and personal fulfillment from being involved in the selection process of students. (29) Hatem et al. reported practical benefits for patients including getting better at finding health care providers and increased knowledge of their medical condition. (40)

The drawbacks and risks associated with patient involvement in medical education included being confronted with stigmatizing assumptions, vulnerability of self-disclosure and spontaneous questions-answer exchanges. A patient educator teaching on the subject of HIV for example described the experience of being very frustrated with one man's lack of knowledge and ignorance about the disease. Patients also drew attention to the fact that unanticipated disease progression had an impact on their ability to teach. In some cases this resulted in them pulling out of their commitment as teachers. inevitable loss among patient educators. (40) Patients also described a sense of vulnerability to negative and non-appreciative reactions from students. (37, 44) Initially, mentors were commonly anxious and unsure about whether what they shared was of benefit to students. (26) Half of the patients involved in the community based intervention in a socio-economic deprived area expressed feelings of anxiety, apprehension or nervousness prior to the interview, although in all cases patients felt that this was normal. (49) In addition to the word ''vulnerable'', patients employed terms like ''exposed'', ''frightened'', ''tired'', ''stressed'' and ''harrowing'' to emphasize service users' feelings within the clinical education environment. (68) Some even described it as traumatic for mental health service users to repeatedly tell their stories. (68)

Financial implications of patient involvement in medical education

Nineteen articles commented on any financial aspect of the interventions, ranging from reimbursement of patients' expenses, payments of honoraria, organizational costs, or perceptions of cost. Economic and financial resources, however, have not been explored in a way that they can be systematically compared. Reported financial costs included $800 for a disability skills workshop (60), £800 for a physical examination training (51), and £2640 for the overall Patient Partners programme (44). Ten studies offered participating patients remuneration through honoraria between €8 per hour and £350 per day (40, 50, 51) or an unspecified amount. (25, 32, 36, 45, 47, 54) Four studies offered reimbursement of patient expenses such as travel, phone or mail costs. (21, 31, 44, 62) Some staff feared that cash patients needed to spend on refreshments or public transport would be a barrier for their participation. (71)

Medical educators suggested that patient involvement was a costly endeavor, both in financial investment as well as staff time. (68) Only one article commented on cost-effectiveness, noting that patient-led teaching is a cost-effective method compared to physician-led teaching, but did not provide an economic evaluation. (51) No paper provided a cost-effectiveness or cost-benefit analysis.

Potential roles of patient organizations in promoting PI in medical education

The most cited interaction between patient organizations and medical faculties was the use of patient organizations and their networks for the recruitment of individual patients as community members or members of condition-specific support groups. (21, 25, 26, 33, 46, 52, 53, 57, 66) In the paper by Baral (2016), representatives from rural communities and consumer groups were consulted by the Medical School Steering Committee for the development of the Academy of Health Sciences curriculum. (42) Representatives of community-based patient advocacy and support organizations took part in the advisory group of the intervention in the study by Towle et al (2013). (46) Not all of these representatives of patient organizations were patients themselves. They were described as brokers between two cultures of academia and community.

The University of Leeds worked with a dedicated internal patient group named "The Patient Voice Group", consisting of lay people who use their experiences to inform their roles as teachers and researchers. This group was involved in formative and summative assessment. Additionally, a Patient and Public Involvement Manager who provided ongoing support was assigned within the school. (36)

Some medical educators made the explicit choice not to collaborate with patient organizations, due to a fear of working with politicized groups. (68) They did make a decision to include patient groups to allow multiple voices to be heard in order to prevent criticisms of tokenism. Patients stated that participating in groups gave them support and companionship from their peers. (68)

Patient organizations wishing to engage in medical education may wish to consider some of the practical points as described in [**BOX 3]**

Measures to ensure the sustainability of patient involvement

The key factors identified in sustaining patient involvement were the provision of adequate resource support, formal acknowledgement of the value of lay contributions, and a clear faculty commitment to change following lay input. (23)

Institution-wide incorporation of social accountability or patient-centered education and medicine in the university's mission and vision statement or strategic plan were cited in several papers to ensure patient and community involvement. Authors emphasized that the resource intensity of patient involvement program requires the university to value its patient-centered underpinnings in order to be sustainable. (23, 41, 42, 58) The incorporation of initiatives as ongoing modules in the curriculum achieved sustainable patient involvement rather than sporadic involvement. (50, 60)

In the university of Leeds, a permanent patient voice group was incorporated in the institution. (36) The institution appointed a Patient and Public Involvement Manager to provide ongoing support. Some initiatives chose to work in partnership with existing institutions, implemented at a school-wide level (42) or focused on one condition such as arthritis. (55) Gaver et al. (2005) identified the process of establishing commitment among volunteering organizations and families as a key challenge to the sustainability of patient involvement. (52)

Medical educators commented that if patient-educators were paid and seen as an employee of the medical school, they might take on the role more seriously and become more reliable, as well as being seen as a respected part of the educational team. (63)

Discussion

This review systematically evaluated 49 primary empirical studies and was aimed at providing updated integrated evidence on the role and impact of the active involvement of patients in medical undergraduate education. The new body of empirical evidence shows the increasing range of learning objectives and educational settings in which patients play an active part in undergraduate medical education. Our review also reveals important information about the potential risks and drawbacks of patient involvement for both students and patient and underlines the need for more structural and integrated patient involvement supported by thorough economic evaluations. Overall the strength of the evidence from the identified studies was low, because most studies were observational or qualitative, and only 7 papers were based on controlled trials.

For peer review only - http://bmjopen.bmj.com/site/about/guidelines.xhtml

The learning objectives identified in this review encompassed all but one of the CanMEDS™ roles for future physicians. This demonstrates that patient's involvement is continuing to gain a larger influence on a diverse range of aspects of the medical curriculum.

Studies described various types of roles for patients, but the vast majority of papers cited the role of a patient teacher. More recent studies suggest that patients are increasingly involved in curriculum development. Most of these initiatives were incidental and were lacking institutional incorporation and longitudinal involvement.

The patients' roles identified in this review are largely in accordance with level 3-6 of the spectrum proposed in the review by Towle et al. (2010). [**BOX 4**] We additionally identified new roles that could not be ascribed to one specific level on this spectrum. In one role, patients did take on roles as equal in curriculum development, but only to specific courses rather than the curriculum as a whole, falling between levels 4 and 5. In another role, patients were consulted in institution level topics and curriculum development beyond specific courses, but rather than being equal partners, they were consulted in a faculty driven initiative, displaying partial elements of level 4, 5 and 6.

Our study found that patients described material, professional, personal and emotional benefits of participating in medical education. Several studies however reported on the potential harms and negative experiences. Concerns related to patient involvement coming from students, faculty and patients themselves should be closely monitored in a systematic manner and addressed appropriately.

An important limitation was the lack of common terminology in the existing literature, potentiating the risk of missing relevant articles, which has been previously reported as a limitation by other review authors. (2)

Our systematic review included only original literature from peer-reviewed journals. As many articles written by patients on their involvement in medical education may only be found in grey literature, including blog posts, conference statements, and patient organization newsletters, this review runs a risk of having missed important aspects of patient views on this topic.

Future research should focus on the long-term effects for patients, students and the healthcare system, especially on the subjects of patient-centeredness and shared decision making. This gap in research limits recommendations that can be made based on current literature. Additionally, no paper performed an economic evaluation of patient involvement, which may be a critical factor for decision makers in educational policy. Finally more research is needed to update existing frameworks for patient involvement to the newly identified roles and needs patients have in medical education.

Conclusion

It has been over 40 years since the first article on patient involvement in medical education was published. Today both the medical education community and the patient community have joined together in the movement to promote patient-centeredness. This systematic review provides knowledge and practical considerations that can aid curriculum developers that wish to sustainably incorporate active patient involvement in their institutions, and patient organizations wishing to engage in medical education.

Contributors

SD and ED jointly developed the review protocol and search strategy, and SD, ED and MW jointly developed the data extraction sheet. The search strategy was critically reviewed by a hospital information specialist. Data collection was carried out by SD and ED and extraction was carried out by SD, ED and MW. The paper was written jointly by all three authors.

Acknowledgements

The authors would like to thank Tessa Richards for her valuable comments and suggestions.

Data sharing

The authors confirm that the data supporting the findings of this study are available within its supplementary materials.

Funding

No funding was obtained for the performance of this review.

For peer review only - http://bmjopen.bmj.com/site/about/guidelines.xhtml

Competing interests

We have read and understood BMJ policy on declaration of interests and declare that we have no competing interests.

Ethical approval

Ethical approval for this study was not applicable, as no human or animal subjects were involved.

Patient and public involvement statement

The initial impulse for the research initiative came was formed by a collaboration between representatives of the International Federation of Medical Students' Associations (IFMSA) and the International Alliance of Patient Organizations (IAPO). MW, co-author, was involved in all stages of data analysis, discussion and writing of the paper. The initial draft was presented and discussed at the European Patient Forum 2019 in a plenary session with over 300 patient representatives present. The authors incorporated the feedback from the European Patient Forum and BMJ's PPI lead, Tessa Richards, into the final draft. The results will be disseminated through the networks of IAPO to reach patient organizations, and will be presented at future patient and medical education conferences.

License

The corresponding author has the right to grant on behalf of all authors and does grant on behalf of all authors, a worldwide licence to the publishers and its licensees in perpetuity, in all forms, formats and media (whether known now or created in the future), to i) publish, reproduce, distribute, display and store the Contribution, ii) translate the Contribution into other languages, create adaptations, reprints, include within collections and create summaries, extracts and/or, abstracts of the Contribution, iii) create any other derivative work(s) based on the Contribution, iv) to exploit all subsidiary rights in the Contribution, v) the inclusion of electronic links from the Contribution to third party material where-ever it may be located; and, vi) licence any third party to do any or all of the above.

Transparency declaration

The lead author affirms that this manuscript is an honest, accurate and transparent account of the study being reported; that no important aspects of the study have been omitted; and that any discrepancies from the study as planned (and, if relevant, registered) have been explained

References

1. Wykurz G, Kelly D. Developing the role of patients as teachers: literature review. BMj. 2002;325(7368):818-21.
2. Towle A, Bainbridge L, Godolphin W, Katz A, Kline C, Lown B, et al. Active patient involvement in the education of health professionals. Medical education. 2010;44(1):64-74.
3. GMC. Patient and public involvement in undergraduate medical education. Advice supplementary to Tomorrow's Doctors (2009). General Medical Council. Great Britain: GMC; 2011.
4. Dijk SW. Meaningful Patient Involvement in Medical Education. Academic medicine-philadelphia-. 2017;92(5):575-81.
5. Boelen C, Heck JE, World Health O. Defining and measuring the social accountability of medical schools. Geneva: World Health Organization, 1995.
6. Towle A, Farrell C, Gaines ME, Godolphin W, John G, Kline C, et al. The patient's voice in health and social care professional education: the Vancouver Statement. International Journal of Health Governance. 2016;21(1):18-25.
7. Department of H. Involving patients and the public in healthcare: A discussion document. Department of Health London; 2001.
8. Spencer J, Blackmore D, Heard S, McCrorie P, McHaffie D, Scherpbier A, et al. Patient-oriented learning: a review of the role of the patient in the education of medical students. Medical education. 2000;34(10):851-7.
9. BMA. Medical Education Subcommittee. Role of the patient in medical education. British Medical Association. 2008.
10. Tew J, Gell C, Foster S. Learning from experience: Involving service users and carers in mental health education and training: University of Nottingham, School of Sociology & Social Policy; 2004.
11. Howe A, Anderson J. Involving patients in medical education. Bmj. 2003;327(7410):326-8.
12. Hasman A, Coulter A, Askham J. Education for partnership. Developments in medical education. 2006.
13. Department of Health. The expert patient: a new approach to chronic disease management for the 21st century. Great Britain: Department of Health; 2001.
14. Organizations IAoP. What is Patient-Centered Healthcare? A Review of Definitions and Principles. London, United Kingdom: IAPO; 2007.
15. Rimmer A. Patients to be asked what they need from doctors. Bmj. 2019;366:l4690.
16. Department of H. The NHS improvement plan: Putting people at the heart of public services: DoH; 2004.
17. Jha V, Setna Z, Al-Hity A, Quinton ND, Roberts TE. Patient involvement in teaching and assessing intimate examination skills: a systematic review. Medical education. 2010;44(4):347-57.
18. Jha V, Quinton ND, Bekker HL, Roberts TE. Strategies and interventions for the involvement of real patients in medical education: a systematic review. Medical education. 2009;43(1):10-20.

For peer review only - http://bmjopen.bmj.com/site/about/guidelines.xhtml

19. Morgan A, Jones D. Perceptions of service user and carer involvement in healthcare education and impact on students' knowledge and practice: a literature review. Medical Teacher. 2009;31(2):82-95.
20. Cooper H, Spencer-Dawe E. Involving service users in interprofessional education narrowing the gap between theory and practice. Journal of Interprofessional Care. 2006;20(6):603-17.
21. Doucet S, Lauckner H, Wells S. Patients' messages as educators in an interprofessional health education program. Journal of Research in Interprofessional Practice and Education. 2013;3(1).
22. Council AM. Assessment and Accreditation of Medical Schools. Standards and procedures. 2002.
23. O'Keefe M, Jones A. Promoting lay participation in medical school curriculum development: lay and faculty perceptions. Medical education. 2007;41(2):130-7.
24. Collins L, Arenson C, Jerpbak C, Kane P, Dressel R, Antony R. Transforming chronic illness care education: A longitudinal interprofessional mentorship curriculum. Journal of Interprofessional Care. 2011;25(3):228-30.
25. Owen C, Reay RE. Consumers as tutors–legitimate teachers? BMC Medical Education. 2004;4(1):14.
26. Towle A, Brown H, Hofley C, Kerston RP, Lyons H, Walsh C. The expert patient as teacher: an interprofessional Health Mentors programme. The Clinical Teacher. 2014;11(4):301-6.
27. Ivory K, Bandler L, Hawke C, Armstrong B. A clinical approach to population medicine. The clinical teacher. 2013;10(2):94-8.
28. McKinlay E, McBain L, Gray B. Teaching and learning about chronic conditions management for undergraduate medical students: utilizing the patient-as-teacher approach. Chronic illness. 2009;5(3):209-18.
29. Stagg P, Rosenthal DR. Why community members want to participate in the selection of students into medical school. Rural & Remote Health. 2012;12(3).
30. Hudson GL, Maar M. Faculty analysis of distributed medical education in Northern Canadian Aboriginal communities. Rural & Remote Health. 2014;14(4).
31. Nestel D, Gray K, Simmons M, Pritchard SA, Islam R, Eng WQ, et al. community perceptions of a rural medical school: a pilot qualitative study. Advances in medical education and practice. 2014;5:407.
32. Fritz C, Naylor K, Watkins Y, Britt T, Hinton L, Curry G, et al. Are we missing the mark? The implementation of community based participatory education in cancer disparities curriculum development. Journal of racial and ethnic health disparities. 2015;2(2):237-43.
33. Noonan EJ, Sawning S, Combs R, Weingartner LA, Martin LJ, Jones VF, et al. Engaging the transgender community to improve medical education and prioritize healthcare initiatives. Teaching and learning in medicine. 2018;30(2):119-32.
34. Kamaka ML. Designing a cultural competency curriculum: asking the stakeholders. Hawaii medical journal. 2010;69(6 Suppl 3):31.
35. Cheng PTM, Towle A. How patient educators help students to learn: an exploratory study. Medical teacher. 2017;39(3):308-14.
36. Muir D, Laxton JC. Experts by experience; the views of service user educators providing feedback on medical students' work based assessments. Nurse education today. 2012;32(2):146-50.
37. Lauckner H, Doucet S, Wells S. Patients as educators: the challenges and benefits of sharing experiences with students. Medical education. 2012;46(10):992-1000.
38. Oswald A, Czupryn J, Wiseman J, Snell L. Patient-centred education: what do students think? Medical education. 2014;48(2):170-80.
39. Oswald AE, Bell MJ, Wiseman J, Snell L. The impact of trained patient educators on musculoskeletal clinical skills attainment in pre-clerkship medical students. BMC medical education. 2011;11(1):65.
40. Hatem DS, Gallagher D, Frankel R. Challenges and opportunities for patients with HIV who educate health professionals. Teaching and learning in medicine. 2003;15(2):98-105.
41. Jarvis-Selinger S, Ho K, Novak Lauscher H, Liman Y, Stacy E, Woollard R, et al. Social accountability in action: University-community collaboration in the development of an interprofessional Aboriginal health elective. Journal of Interprofessional Care. 2008;22(sup1):61-72.
42. Baral K, Allison J, Upadhyay S, Bhandary S, Shrestha S, Renouf T. Rural Community as Context and Teacher for Health Professions Education. Cureus. 2016;8(11).
43. Baral KP, Upadhyay SK, Bhandhary S, Gongal RN, Karki A. Development of community based learning and education system within undergraduate medical curriculum of Patan Academy of Health Sciences. Journal of Nepal Health Research Council. 2016.
44. Haq I, Fuller J, Dacre J. The use of patient partners with back pain to teach undergraduate medical students. Rheumatology. 2005;45(4):430-4.
45. Duggan A, Bradshaw YS, Carroll SE, Rattigan SH, Altman W. What can I learn from this interaction? A qualitative analysis of medical student self-reflection and learning in a standardized patient exercise about disability. Journal of Health Communication. 2009;14(8):797-811.
46. Towle A, Godolphin W. Patients as educators: interprofessional learning for patient-centred care. Medical teacher. 2013;35(3):219-25.
47. Dogra N, Anderson J, Edwards R, Cavendish S. Service user perspectives about their roles in undergraduate medical training about mental health. Medical Teacher. 2008;30(6):e152-e6.
48. Lazarus PA. Patients' experiences and perceptions of medical student candidates sitting a finals examination. Medical Teacher. 2007;29(5):484-9.
49. Jackson A, Blaxter L, Lewando-Hundt G. Participating in medical education: views of patients and carers living in deprived communities. Medical Education. 2003;37(6):532-8.
50. Bokken L, Rethans J-J, Jöbsis Q, Duvivier R, Scherpbier A, van der Vleuten C. Instructiveness of real patients and simulated patients in undergraduate medical education: a randomized experiment. Academic Medicine. 2010;85(1):148-54.
51. Raj N, Badcock LJ, Brown GA, Deighton CM, O'Reilly SC. Undergraduate musculoskeletal examination teaching by trained patient educators—a comparison with doctor-led teaching. Rheumatology. 2006;45(11):1404-8.
52. Gaver A, Borkan JM, Weingarten MA. Illness in context and families as teachers: a year-long project for medical students. Academic Medicine. 2005;80(5):448-51.
53. Bideau M, Guerne P-A, Bianchi M-P, Huber P. Benefits of a programme taking advantage of patient-instructors to teach and assess musculoskeletal skills in medical students. Annals of the rheumatic diseases. 2006;65(12):1626-30.
54. Jain S, Foster E, Biery N, Boyle V. Patients with disabilities as teachers. Fam Med. 2013;45(1):37-9.
55. Oswald AE, Wiseman J, Bell MJ, Snell L. Musculoskeletal examination teaching by patients versus physicians: how are they different? Neither better nor worse, but complementary. Medical teacher. 2011;33(5):e227-e35.

For peer review only - http://bmjopen.bmj.com/site/about/guidelines.xhtml

56. de Boer A, Melchers D, Vink S, Dekker F, Beaart L, de Jong Z. Real patient learning integrated in a preclinical block musculoskeletal disorders. Does it make a difference? Clinical rheumatology. 2011;30(8):1029-37.
57. Jaworsky D, Gardner S, Thorne JG, Sharma M, McNaughton N, Paddock S, et al. The role of people living with HIV as patient instructors–reducing stigma and improving interest around HIV care among medical students. AIDS care. 2017;29(4):524-31.
58. Barr J, Ogden K, Rooney K. Committing to patient-centred medical education. The clinical teacher. 2014;11(7):503-6.
59. Humphrey-Murto S, Smith CD, Touchie C, Wood TC. Teaching the musculoskeletal examination: are patient educators as effective as rheumatology faculty? Teaching and learning in medicine. 2004;16(2):175-80.
60. Saketkoo L, Anderson D, Rice J, Rogan A, Lazarus CJ. DEVELOPMENTS: Effects of a Disability Awareness and Skills Training Workshop on Senior Medical Students as Assessed with Self Ratings and Performance on a Standardized Patient Case. Teaching and Learning in Medicine. 2004;16(4):345-54.
61. Langlois S, Lymer E. Learning professional ethics: Student experiences in a health mentor program. Education for Health. 2016;29(1):10.
62. Coret A, Boyd K, Hobbs K, Zazulak J, McConnell M. Patient Narratives as a Teaching Tool: A Pilot Study of First-Year Medical Students and Patient Educators Affected by Intellectual/Developmental Disabilities. Teaching and learning in medicine. 2018;30(3):317-27.
63. Jha V, Quinton ND, Bekker HL, Roberts TE. What educators and students really think about using patients as teachers in medical education: a qualitative study. Medical education. 2009;43(5):449-56.
64. Henriksen A-H, Ringsted C. Medical students' learning from patient-led teaching: experiential versus biomedical knowledge. Advances in Health Sciences Education. 2014;19(1):7-17.
65. Alahlafi A, Burge S. What should undergraduate medical students know about psoriasis? Involving patients in curriculum development: modified Delphi technique. Bmj. 2005;330(7492):633-6.
66. Tierney WM. Use of Stakeholder Focus Groups to Define the Mission and Scope of a new Department of Population Health. Journal of general internal medicine. 2018;33(7):1069-76.
67. Wagner AC, Hart TA, McShane KE, Margolese S, Girard TA. Health care provider attitudes and beliefs about people living with HIV: Initial validation of the health care provider HIV/AIDS Stigma Scale (HPASS). AIDS and Behavior. 2014;18(12):2397-408.
68. Rees CE, Knight LV, Wilkinson CE. "User involvement is a sine qua non, almost, in medical education": learning with rather than just about health and social care service users. Advances in Health Sciences Education. 2007;12(3):359-90.
69. Frank JR, Snell L, Sherbino J, Boucher A. CanMEDS 2015. Physician Competency Framework Series I. 2015.
70. Lucas B, Pearson D. Patient perceptions of their role in undergraduate medical education within a primary care teaching practice. Education for Primary Care. 2012;23(4):277-85.
71. Gutteridge R, Dobbins K. Service user and carer involvement in learning and teaching: A faculty of health staff perspective. Nurse education today. 2010;30(6):509-14.

Box 1: Identified patient roles in medical education

Role	Specification
**Patient Teacher**	Deliver clinical skills sessions on history taking, counseling and physical examination<br>Deliver formative feedback during teaching sessions<br>Share experiences in healthcare or personal aspects of their lives in teaching sessions, small group sessions, individual mentorship and coaching, or through the creation of videos
**Patient Assessor**	Deliver formative feedback during teaching sessions<br>Perform summative assessments during OSCE's
**Patient Curriculum developer**	Evaluate the medical program<br>Act as a curriculum steering committee member<br>Were consulted to provide recommendations on disease specific courses through focus groups, world cafés or discussions with community leaders<br>Participate in development of courses related to their illness or social conditions, or overarching competencies and community based learning<br>Develop courses delivered by patient teachers autonomously<br>Identify end-competencies for graduates<br>Identify desired curriculum characteristics<br>Be consulted on the strategic development of a new medical school department
**Patient selection committee member**	Participate in the selection of students applying for the medical program<br>Assess candidates' communication skills, sensitivity, compassion and empathy towards societal contexts and needs.

Box 2: Aspired learning objectives for medical students based on the CANMEDS™ framework

CANMEDS role	Specification
**Communicator**	Apply a patient-centered approach to interviewing and care<br>Adopt to the unique needs and preferences of each patient as an individual, recognizing their needs.<br>Communicate using a patient-centered approach that encourages patient trust and autonomy, recognizing their expertise in care and seeing them as part of a team.<br>Create an environment for patient comfort, dignity, privacy, engagement and safety by using a non-patronizing and nonjudgmental attitude, recognizing biases.<br>Apply communication skills to share information and explanations that are clear and accurate, checking for understanding, using communication skills that help patients make informed decisions
**Collaborator**	Work effectively with physicians and colleagues in the health care professions through interdisciplinary teams

For peer review only - http://bmjopen.bmj.com/site/about/guidelines.xhtml

**Leader**	Contribute to the improvement of health care delivery through understanding the broader health care system, and how it affects patients.
**Professional**	Demonstrate a commitment to patients and society, by applying best practices and adhering high ethical standards, dealing with ethical complexity of clinical practice.<br>Demonstrating a commitment to the profession, reflecting on role models and professional identity, keeping fellow physicians and oneself to high professional standards, and understanding patient views on clinical errors<br>Demonstrating commitment to physician health and well-being by learning to cope with uncertainties, emotions and stress<br>Exhibit appropriate professional behaviors and relationships, demonstrating respect for diversity, respect and compassion.
**Health Advocate**	Responding to individual patient's health needs by advocating with the patient within and beyond the clinical environment, specifically for patients in vulnerable situations.<br>Awareness of the importance of physician and patient advocacy<br>Working with communities or patients to identify determinants of health that affect them
**Scholar**	No paper explicitly described the aim of developing the role of scholar
**Medical Expert**	Performing patient-centered clinical assessments and establishing a management plans<br>Establishing plans for ongoing, continuity of care.<br>Understanding the complexity of practicing medicine<br>Integration of theory into practice

Box 3: Practical points for patient organizations wishing to engage in medical education

Practical points for patient organizations
♦ Highlight potential benefits of involvement in medical education to members and respond to common motivations<br>♦ Facilitate peer support networks among patients involved in medical education<br>♦ Investigate whether teaching facilities in your area have existing frameworks for patients and public involvement, have made explicit commitments to patient and public involvement and/or have appointed officers specifically assigned to patient and public involvement that could provide you with a starting point for collaboration<br>♦ Call for the creation of a patient committee or advisory group within a medical faculty to enable and safeguard structural input into the educational process<br>♦ Determine which step of the educational process is best suited for your goals. You might choose to focus your efforts on the mission and vision of the school, learning objectives, educational strategies, teaching, assessment of learners or the evaluation of the course.<br>♦ Not every meaningful intervention has to be at an overall faculty level, they may also be on specific areas such as one learning objective or a disease-specific course.<br>♦ Showcase successful collaborations with universities and best practices to the wider patient and academic communities

Box 4: Spectrum of patient involvement in medical education by Towle et al. (2010)

Spectrum of patient involvement in medical education as defined by Towle et al. (2010)
1. Patients as focus of a paper-based or electronic cases<br>2. Standardized or volunteer patients in clinical settings<br>3. Patients sharing their experiences with students in a faculty directed curriculum<br>4. Patient-teachers involved in teaching or evaluating students<br>5. Patient-teachers as equal partners in student education, evaluation and curriculum development beyond specific courses, to the curriculum as a whole<br>6. Patients involved at the institutional level in addition to sustained involvement as patient-teachers in education, evaluation and curriculum development.

SUPPLEMENTARY FILE 1: Full search strategy

SUPPLEMENTARY FILE 2: Study characteristics

FIGURE 1: Results of the systematic literature search

For peer review only - http://bmjopen.bmj.com/site/about/guidelines.xhtml

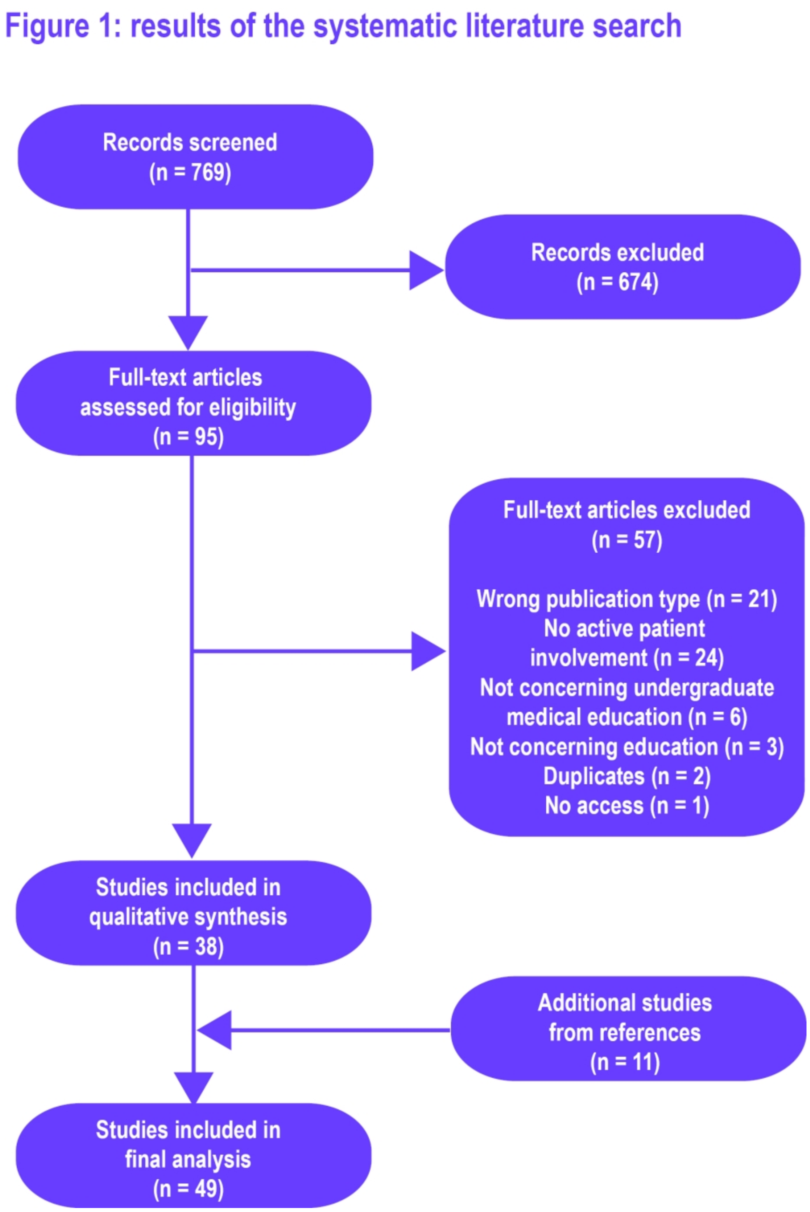

Figure 1: Study flowchart

139x207mm (300 x 300 DPI)

Full search strategy

The authors performed a search through PubMed on July 12th 2018. The following search terms were used:

((“Patient Participation”[Mesh] OR "Community Participation"[Mesh] OR “Stakeholder Participation”[Mesh] OR “patient instructors”[tiab] OR “patient educators”[tiab] OR “patient teachers”[tiab] OR “patient-as-teacher”[tiab] OR patient participation [tiab] OR patient involvement [tiab] OR community participation [tiab] OR community involvement [tiab] OR patient engag [tiab] OR community engag* [tiab] OR patient cooperat* [tiab] OR community cooper* [tiab] OR patient collaborat* [tiab] OR community collaborat* [tiab] OR patient represent* [tiab] OR community represent* [tiab])*

AND

(“Education, Medical, Undergraduate”[Mesh] OR "Education, Medical"[Mesh] OR “Curriculum”[Mesh] OR “Students, Medical”[Mesh] OR medical education [tiab] OR medical student [tiab]))*

A limit was set to only show articles written in English and published between 2003 and 2018 to be included. Exclusion criteria were studies that did not address education to undergraduate medical students, where patients were only passively involved in education (for example, through presence at wards). Articles were first assessed based on title and abstract, and in a second round on full text. The references of all included articles were assessed for additional studies that matched the original inclusion criteria. All reviews that complied with the inclusion criteria were additionally assessed for relevant references, however, only original research articles were included.

For peer review only

Authors	Year of publication	Journal	Title	Country
Rees, C.E., Knight, L.V., Wilkinson, C.E.	2006	Advances in Health Sciences Education	‘‘User Involvement Is a Sine Qua Non, Almost, in Medical Education’’: Learning with Rather than Just About Health and Social Care Service Users	UK
Ivory, K. et al.	2013	The Clinical Teacher	A clinical approach to population medicine	Australia
Fritz, C. et al.	2015	Journal of Racial and Ethnic Health Disparities	Are We Missing the Mark? The Implementation of Community Based Participatory Education in Cancer Disparities Curriculum Development	USA
Bideau, M et al.	2006	Annals of the rheumatic diseases	Benefits of a programme taking advantage of patient-instructors to teach and assess musculoskeletal skills in medical students.	Switzerland
Hatem, D.S., Gallagher, D., Frankel, R.	2003	Teaching and Learning in Medicine	Challenges and Opportunities for Patients With HIV Who Educate Health Professionals	USA
Barr, J. et al.	2014	The Clinical Teacher	Committing to patient-centred education	Australia
Nestel, D. et al.	2014	Advances in Medical Education and Practice	Community percpetions of a rural medical school: a pilot qualitative study	Australia
Owen, C., Reay, R.	2004	BMC Medical Education	Consumers as tutors - legitimate teachers?	Australia
Kamaka, M.L.	2010	Hawai'i Medical Journal	Designing a Cultural Competency Curriculum: Asking the Stakeholders	USA
Baral, K.P., et al.	2016	Journal of Nepal Health Research Council	Development of Community Based Learning and Education system within Undergraduate Medical Curriculum of Patan Academy of Health Sciences	Nepal
Saketkoo, L. et al.	2004	Teaching and Learning in Medicine	Effects of a disability awareness and skills training workshop on senior medical students as assessed with self ratings and performance on a standardized patient case	USA
Noonan E.J. et al.	2018	Teaching and learning in medicine.	Engaging the Transgender Community to Improve Medical Education and Prioritize Healthcare Initiatives	USA
Muir, D., Laxton, J.C.	2012	Nurse Education Today	Experts by experience; the views of service user educators providing feedback on medical students' work based assessments	UK

Hudson, G.L., Maar, M.	2014	Rural and Remote Health	Faculty analysis of distributed medical education in Northern Canadian Aboriginal communities	Canada
Cheng, P., Towle, A.	2017	Medical Teacher	How patient educators help students to learn: An exploratory study	Canada
Gaver, A. et al.	2005	Academic Medicine	Illness in context and families as teachers: A year-long project for medical students	Israel
Bokken, L. et al.	2010	Academic Medicine	Instructiveness of real patients and simulated patients in undergraduate medical education: a randomized experiment	Netherlands
Cooper Director, H., Spencer-Daw, E.	2006	Journal of Interprofessional Care	Involving service users in interprofessional education narrowing the gap between theory and practice	UK
Langlois, S., Lymer, E.	2016	Education for Health	Learning professional ethics: Student experiences in a health mentor program	Canada
Henriksen, A., Ringsted, C.	2014	Advances in Health Sciences Education: Theory and Practice	Medical students' learning from patient-led teaching: experiential versus biomedical knowledge	Denmark
Oswald, A.E. et al.	2011 b	Medical Teacher	Musculoskeletal examination teaching by patients versus physicians: how are they different? Neither better nor worse, but complementary	Canada
Jackson, A., Blaxter, L., Lewando-Hundt, G.	2003	Medical Education	Participating in medical education: views of patients and carers living in deprived communities	UK
Coret, A. et al.	2018	Teaching and Learning in Medicine	Patient Narratives as a Teaching Tool: A Pilot Study of First-Year Medical Students and Patient Educators Affected by Intellectual/Developmental Disabilities	Canada
Lucas, B, Pearson, D.	2012	Education for Primary Care	Patient perceptions of their role in undergraduate medical education within a primary care teaching practice	UK
Oswald, A.E. et al.	2014	Medical Education	Patient-centred education: what do students think?	Canada
Towle, A., Godolphin, W.	2013	Medical Teacher	Patients as educators: Interprofessional learning for patient-centred care	Canada
Lauckner, H., Doucet, S., Wells, S.	2012	Medical Education	Patients as educators: the challenges and benefits of sharing experiences with students	Canada

Jain, S. et al.	2013	Family Medicine	Patients with Disabilities as Teachers	USA
Lazarus, P.A.	2007	Medical Teacher	Patients' experiences and perceptions of medical student candidates sitting a finals examination	UK
Doucet, S., Lauckner, H., Wells, S.	2013	Journal of Research in Interprofessional Practice and Education	Patients' Messages as Educators in an Interprofessional Health Education Program	Canada
O'Keefe, M., Jones, A.	2007	Medical Education	Promoting lay participation in medical school curriculum development: lay and faculty perceptions	Australia and NZ
de Boer, A. et al.	2011	Clinical Rheumatology	Real patient learning integrated into a preclinical block musculosceletal disorders. Does it make a difference?	Netherlands
Baral, K. et al.	2016	Cureus	Rural Community as Context and Teacher for Health Professions Education	Nepal
Gutteridge, R., Dobbins, K.	2009	Nurse Education Today	Service user and carer involvement in learning and teaching: A faculty of health staff perspective	UK
Dogra, N. et al.	2008	Medical Teacher	Service user perspectives about their roles in undergraduate medical training about mental health	UK
Jarvis-Selinger, S. et al.	2008	Journal of Interprofessional Care	Social accountability in action: University-community collaboration in the development of an interprofessional Aboriginal health elective	Canada
McKinley, E, McBain, L., Gray, B.	2009	Chronic Illness	Teaching and learning about chronic conditions management for undergraduate medical students: utilizing the patient-as-teacher approach	NZ
Humphrey-Murto, S. et al.	2004	Teaching and Learning in Medicine	Teaching the Musculoskeletal Examination: Are Patient Educators as effective as Rheumatology Faculty?	Canada
Towle, A. et al.	2014	The Clinical Teacher	The expert patient as teacher: an interprofessional Health Mentors programme.	Canada
Oswald, A.E. e.al.	2011 a	BMC Medical Education	The impact of trained patient educators on musculoskeletal clinical skills attainment in pre-clerkship medical students	Canada
Jaworsky, D. et al.	2017	AIDS Care	The role of people living with HIV as patient instructors – reducing stigma and improving interest around HIV care among medical students	Canada

Haq, I. Fuller, J., Dacre, J.	2006	Rheumatology	The use of patient partners with back pain to teach undergraduate medical students	UK
Collins, L. et al.	2011	Journal of Interprofessional Care	Transforming chronic illness care education: A longitudinal interprofessional mentorship curriculum	USA
Raj, N. et al.	2006	Rheumatology	Undergraduate musculoskeletal examination teaching by trained patient educators—a comparison with doctor-led teaching	UK
Tierney, W.M.	2018	Journal of General Internal Medicine	Use of Stakeholder Focus Groups to Define the Mission and Scope of a new Department of Population Health.	USA
Duggan, A. et al.	2009	Journal of Health Communication	What can I learn from this interaction? A qualitative analysis of medical student self-reflection and learning in a standardized patient exercise about disability	USA
Jha, V. et al.	2009	Medical Education	What educators and students really think about using patients as teachers in medical education: a qualitative study	UK
Alahlafi, A., Burge, S.	2005	British Medical Journal	What should undergraduate medical students know about psoriasis? Involving patients in curriculum development: modified Delphi technique	UK
Stagg, P., Rosenthal, D.R.	2012	Rural and Remote Health	Why community members want to participate in the selection of students into medical school	Australia

Methods used	Research Focus	Outcome measurement	Number of patients involved in intervention (if
Focus group discussions analysed using Framework Analysis	Service user involvement, patients as teachers, socio-cultural learning	Service user views and experiences	19 service users, 47 stakeholders in total
Report on programme development	Development of an integrated, community-engaged population medicine curriculum	Evaluation of stakeholder experiences	
Deliberative session, quantitative and qualitative survey	Community interests in health disparities curriculum design	Patient perspectives on creation of a cancer disparities curriculum for medical students and public health students	86
Pre- and post-intervention questtionaires and direct performance assessment	Evaluate ability of a patient-instructor program to improve and assess musculoskeletal knowledge and skills	Improved observed and reported student performance	11
Semistructured interviews with patient-faculty	Decision to teach, and personal, medical and psychosocial consequences	Patient motivation, experiences and changes in relationships	8
Summary report on eight years of programme	Patient Partner Program, as a traning platform for communication and consultative skills research	Description of the program, implications, impact on learning, challenges	
Telephone interviews	Community interests in curriculum development	Expectations of lay community members	12 community members
Programme evaluation	Psychiatry curriculum, consumer perspective, patient educators	Patient, student and tutor feedback on experience and attitudes	20 received training, after 4 years 15 remain.
Focus group discussionswith native hawaiian stakeholders	Development of a curriculum that addresses native hawaiian health and cultural competencies	Themes resulting from community focus grous	34 stakeholders (patients, medical students and physiicians)
Literature review and series of consultative meetngs with stakeholders	Curriculum development process for the community based education	Framework forcurriculum development	
Project report	Workshop on medical student disability skills and awareness	Student self-efficacy ratings, knowledge of disability resources and medical complications, attitudes, confidence in ability to effectively advocate for patients	2 patients 1 parent
Forum discussion and follow up survey	Transgender health, Intergroup contact theory	LGBT community views on transgender disparities in medical education	10-15 Transgender participants
Analysis of student assessments by patient group	The use of interprofessional assessment tool by patients	Reflections by patient assessors	6 (of which 3 took place in a focus group at a later stage)

Report on pilot project	Development of an Aboriginal health curriculum	Lessons learned and suggestions for curriculum improvement	7 Aboriginal communities
Exploratory study	Interprofessional Health Mentors Program	Mentor experiences and teaching methods used	11 Mentors
Report on project	Family-centered care	Student learning based on analysis of essays	100 (volunteer families)
Controlled study	Comparison between simulated patients and. real patients	Instructiveness to students (quantitative and qualitative)	9 real patients, 5 simulated patients
Evaluation report on pilot project	Interprofessional Education (IPE), Complexity Theory	Effects of IPE on theory practice gap of service users, students and facilitators	35, of which 14 were included for in-depth interviews
Report on pilot project	Interprofessional education, medical ethics, patient-centredness, Chronic Health	Student experiences and learning	17
Focus groups	Patient-centredness, shared decision making and patient empowerment, arthritis educator programme	Student perceptions	22
Comparative study	Patient educators (Patient Partners) compared to standardized patients	Observed qualitative comparison of features and themes emerging from training	2 patient tutors
Interviews	Patient views on participation in medical education	Patient and carers views	54
Controlled pilot study	Report on study about educational experience, communication skills assessment and attitudes assessment	Qualitative and quantitative observed and self-reported data on student performance in clinical encounters	4 patient case videos and 3 caregiver videos followed by encounters with
In-depth interviews	Patient perceptions of their role in undergraduate medical education within primary care	Patient perceptions	18 patients, 5 female, 13 male
Analysis of written student assignments and focus group discussions	Student reflections on experiences of patient-educators	Student reflections on experiences of patient-educators	
Report on evaluation of ongoing programme	Physician-patient communication, service user involvement, Interprofessional Education (IPE), chronic care, patient-centredness	Workshop design, student experiences and learning outcomes	24 Community Educators
Semi-structured focus group discussions and individual interviews	Dalhousie Health Mentors Programme	Patient educator experiences	30

Programme evaluation	Health communication, relationship-centred care and patient-centred medical home	Students experience and suggestions	2
Group or individual interviews	Improving training of medical students based on patient assessment	Patient perceptions and opinons of students during final examinatin	10
Semi-structured focus groups and individual interviews	Interprofessional Education, Dalhousie Health Mentors Programme	Mentor feedback to the program	30
Focus groups	Consumer and community participation in curriculum development	Identiication of challenges of lay participation in medical curriculum development	17
Report on pilot project	Preclinical Real Patient Learning in musculoskeletal disorders	Educational effectiveness, student opinions and expectations, experienced effects	>4
Progress report	Describing an approach of ruralizing the academic medical education	Description of the program	
Analysis of interviews, quality reports, university policy documents, programme specifications and model descriptors	Barriers to effective patient involvement	Academic and administrative staff views	
Focus groups	Service user perspectives on their role in delivery of teaching psychiatry	Service user focus group feedback and suggestions	28
Report on pilot project	Co-development of an Aboriginal Health course	Recommendations for curriculum development, issues and experiences	2 Aboriginal communities
Qualitative questionnaire	Impact of student learning of chronic conditions teaching programme utilizing patients-as-teachers	Student feedback on involvement of patients in teaching/home visit (before/after)	
Randomized comparative study	Comparison of effectiveness of patients as educators and rheumatology faculty using musculoskeletal examination	Student test results for skills (nine station OSCE) and feedback	10 (5 educators and 5 silent patients)
Report on evaluation of ongoing programme	Interprofessional Education (IPE), Health Mentors Programme	Student and patient experiences, implications for programme development	
Randomized, controlled, cross-over designed cohort study	Patients as educators in muskuloskeletal disease compared to non-specialist physician tutors	Student exam results and experience ratings	
Report on pilot project	Pre- and post simulated clinical encounter HIV-related stigma	Stigma scale HPASS, interest and comfort in providing HIV care	16

Report on pilot project	Patients as educators (Patient Partners)	Student OSCE results, experience. Patient Partner experiences	4
Program evaluation report	Interprofessional Health Mentors Program and Chronic Care Model	Student perspectives on the programme and chronic illness	
Randomized comparative study	Comparison between Patient Educators and Doctors in teaching hand and knee examination	Student OSCE scores	12 patient educators trained (6 teach)
Focus groups	Input for strategic development of department of population health in new medical school	Consensus on focus areas for the school's curriculum	13 community members
Qualiative analysis of self-reflections	Disability health care, Consumer Protection, Standardized Patient Educators, relationship-centered care	Student reflections and insights gained	12
Cross-sectional survey	Patients as teachers, UK Department of Health	Student and medical tutor views and experiences on patients as teachers in medical training	
Delphi study	To identify the content of a psoriasis curriculum for medical students	Consensus among patients/physicians/nurses on content of the psoriasis workshop.	20 patients
Interviews	Community member participation in selection of medical students	Patient experience and perceptions	8 community members

For peer review only - http://bmjopen.bmj.com/site/about/guidelines.xhtml

Patient characteristics	Role of patient organizations
Ages 20-86, majority white. Other stakeholders were medical students and medical educators.	Medical educators explicitly preferred not to work with politicized groups of patients, but did include groups to hear multiple voices. Service users wanted to participate in groups in order to receive support.
Patients with chronic health conditions	Partnership with consumer organizations to recruit patients
Members of Chicago community (focus on cancer)	Study concept directly derived from cancer support group
All women, aged 29–66 years, who have had rheumatoid arthritis for at least 5 years.	Involved in patient recruitment
People living with HIV/AIDS, age 26-50	
Patients from community practices (specialist and general)	
Any member of lay public, recruited through local newspaper advertisement	
Current consumer of mental health services	Partner in steering group and recruitment
Native Hawai'ians	
Community members including heads of households hosting students during field stay, local community leaders and local health workers are involved in assessing the students' behaviors during field postings.	
1 team member with visual impairment, 1 attorney with mental illness, 1 parent of a nonverbal child	Partner in organizing team
Transgender	Recruitment via local LGBT organizations
Lay people with experiences of ill health, caring and accessing health services	Lay group "The Patient Voice Group" of 6 members participated in the project

Aboriginal communities	
Various conditions, 30-60 years	
Volunteer family, one of whose members suffers from a chronic disease or other impairment.	Involved in recruitment of families
Patients with asthma (age 54-82)	
Health or social care service users	Advise, provide assistance and support
People with chronic health challenges	
Patient Instructors associated with Danish Association of Rheumatism	Employer of Patient Instructor leader
Lay people with a chronic MSK condition who undertake formal training in teaching musculo-skeletal physical examination skills	
Patients who live in socially and economically deprived areas, child carers, midlife patients and older adult patients	
People living with intellectual and developmental disabilities (IDD)	
Patients who volunteered for involvement with pre-arranged undergraduate clinics	
Volunteer patient educators with arthritis	
Community Educators with epilepsy, arthritis, HIV/AIDS and mental health problems	Community organizations with a health mandate are part of Advisory Group
Adult volunteers with chronic conditions or disabilities. Mentors' conditions included diabetes, chronic pain, multiple sclerosis, cancer, stroke and various rare conditions.	

Persons with disabilities in their late 20's, who use wheelchairs; 1. spinal musclar atrophy, 1 cerebral palsy.	
Patients, who had been involved in final examns, all >30 y.o, all white British ethnicity	
Community volunteers with a chronic condition and/or disability	Recruitment via patient networks
Lay public	
Patients of the departments of rheumatology, orthopedic surgery, and/or patients under treatment of the local physical therapists.	Patients with spondylarthritis are members of a professional organization of arthritis educators which employs patients with RA in different phases of their disease.
Community members and leaders from rural areas, women's groups, CBOs, red cross, local health institutions	
16 men 12 women, all white adults, one of them a carer	
Aboriginal leaders, instructors and Elders	
Patients with a specific, usually physical, chronic condition (such as diabetes)	
5 patient educators and 5 silent patients; patients with arthritis	
Patients with a wide range of conditions, including HIV/AIDS, arthritis, multiple sclerosis, spinal cord injury, cerebral palsy and mental health problems	Community organizations facilitate mentor recruitment
Volunteer patient educators with arthritis	
People living with HIV/AIDS	PHAs were recruited through a community partner, the Toronto People With AIDS Foundation

Three male patients and one female , age 29–56. Ankylosing spondylitis and chronic back pain with intermittent sciatica.	
Patients with chronic illness or disability	
11 female, 1 male, age 34–79. Rheumatoid arthritis, osteoarthritis, inflammatory arthritis.	
Local community non-profit organizations	People recruited from local community organizations
Patients with an apperant disability; included Multiple Sclerosis, Juvinile rheumathoid arthritis, traumatic spinal cord injury, neurodegenerative disorders, cerebral palsy, muscular dystrophy, blindness, paraplegia, quadriparesis, respiratory failure, NF and mobility constraint	
Patients with psoriasis	
Community Liaison Committee members	

ew only

Type of patient engagement	Number of students involved in
Provide feedback on programme	
Curriculum development	
Active teacher, student assessor, feedback/coach	61
Curriculum development, development of learning material, active teacher	
Share experiences, give feedback on students' interviewing skills	
Interviewed to share perspective on community engagement	
Active patient teacher, curriculum development	104
Curriculum development	4, 1 male, 3 female
Feedback/coach; student selection, teacher, assessor	
Active teacher	147
Curriculum development	
curriculum development, development of learning material, active teacher, student assessor, feedback/coach	253

For peer review only

Curriculum development, student assessor, feedback/coach, student selection	15 medical and nursing students
Active teacher "The health mentor is the primary teacher; faculty are not present in the meetings but provide background support through setting broad topics and responding to students' reflections written	
Active teacher	40 randomly selected student essays
Active teacher, feedback/coach	163 (61 in real patient group and 102 in simulated patient group)
Curriculum development, active teacher	22 groups of approximately 10 students (of which 63 were analysed for the
Active teacher, Feedback/coach	91
Curriculum development, development of learning material, active teacher	Of 39 students enrolled in programme, 23 were included in the focusgroups
Active teacher	6 small group sessions
Active teacher	
Narrator	27, 23 female
Active teacher	
Active teacher	356
Curriculum development, development of learning material, active teacher	142
Active teacher	

For peer review only

Active teacher	44
Curriculum development and student assessor	
Active teacher	
Curriculum development	
Active teacher	377
Curriculum development, selection of students, setting of school mission and vision, teaching	
Curriculum development	
Curriculum development	7
Active teacher	57
Active teacher	62
Active teacher, mentorship/coach	290
Active teacher	83
Active teacher	67

For peer review only

Active teacher	60
Coach/mentorship	60 essays analyzed (out of 270 papers collected)
Active teacher	50
Curriculum development	
Standardized Patient Educator	138
Curriculum development, active teacher, assessor	28
Curriculum development	
Student selection	

For peer review only

PRISMA 2009 Checklist

Section/topic	#	Checklist item	Reported on page #
**TITLE**			
Title	1	Identify the report as a systematic review, meta-analysis, or both.	1
**ABSTRACT**			
Structured summary	2	Provide a structured summary including, as applicable: background; objectives; data sources; study eligibility criteria, participants, and interventions; study appraisal and synthesis methods; results; limitations; conclusions and implications of key findings; systematic review registration number.	2
**INTRODUCTION**			
Rationale	3	Describe the rationale for the review in the context of what is already known.	3
Objectives	4	Provide an explicit statement of questions being addressed with reference to participants, interventions, comparisons, outcomes, and study design (PICOS).	3
**METHODS**			
Protocol and registration	5	Indicate if a review protocol exists, if and where it can be accessed (e.g., Web address), and, if available, provide registration information including registration number.	3
Eligibility criteria	6	Specify study characteristics (e.g., PICOS, length of follow-up) and report characteristics (e.g., years considered, language, publication status) used as criteria for eligibility, giving rationale.	3
Information sources	7	Describe all information sources (e.g., databases with dates of coverage, contact with study authors to identify additional studies) in the search and date last searched.	3
Search	8	Present full electronic search strategy for at least one database, including any limits used, such that it could be repeated.	3, Supplementary
Study selection	9	State the process for selecting studies (i.e., screening, eligibility, included in systematic review, and, if applicable, included in the meta-analysis).	3
Data collection process	10	Describe method of data extraction from reports (e.g., piloted forms, independently, in duplicate) and any processes for obtaining and confirming data from investigators.	3
Data items	11	List and define all variables for which data were sought (e.g., PICOS, funding sources) and any assumptions and simplifications made.	-
Risk of bias in individual studies	12	Describe methods used for assessing risk of bias of individual studies (including specification of whether this was done at the study or outcome level), and how this information is to be used in any data synthesis.	-
Summary measures	13	State the principal summary measures (e.g., risk ratio, difference in means).	-
Synthesis of results	14	Describe the methods of handling data and combining results of studies, if done, including measures of consistency (e.g., I^2) for each meta-analysis.	3

For peer review only - http://bmjopen.bmj.com/site/about/guidelines.xhtml

PRISMA 2009 Checklist

Section/topic	#	Checklist item	Reported on page #
Risk of bias across studies	15	Specify any assessment of risk of bias that may affect the cumulative evidence (e.g., publication bias, selective reporting within studies).	-
Additional analyses	16	Describe methods of additional analyses (e.g., sensitivity or subgroup analyses, meta-regression), if done, indicating which were pre-specified.	-
**RESULTS**			
Study selection	17	Give numbers of studies screened, assessed for eligibility, and included in the review, with reasons for exclusions at each stage, ideally with a flow diagram.	3,4
Study characteristics	18	For each study, present characteristics for which data were extracted (e.g., study size, PICOS, follow-up period) and provide the citations.	Supplementary
Risk of bias within studies	19	Present data on risk of bias of each study and, if available, any outcome level assessment (see item 12).	-
Results of individual studies	20	For all outcomes considered (benefits or harms), present, for each study: (a) simple summary data for each intervention group (b) effect estimates and confidence intervals, ideally with a forest plot.	-
Synthesis of results	21	Present results of each meta-analysis done, including confidence intervals and measures of consistency.	-
Risk of bias across studies	22	Present results of any assessment of risk of bias across studies (see Item 15).	-
Additional analysis	23	Give results of additional analyses, if done (e.g., sensitivity or subgroup analyses, meta-regression [see Item 16]).	-
**DISCUSSION**			
Summary of evidence	24	Summarize the main findings including the strength of evidence for each main outcome; consider their relevance to key groups (e.g., healthcare providers, users, and policy makers).	9
Limitations	25	Discuss limitations at study and outcome level (e.g., risk of bias), and at review-level (e.g., incomplete retrieval of identified research, reporting bias).	10
Conclusions	26	Provide a general interpretation of the results in the context of other evidence, and implications for future research.	10
**FUNDING**			
Funding	27	Describe sources of funding for the systematic review and other support (e.g., supply of data); role of funders for the systematic review.	10

From: Moher D, Liberati A, Tetzlaff J, Altman DG, The PRISMA Group (2009). Preferred Reporting Items for Systematic Reviews and Meta-Analyses: The PRISMA Statement. PLoS Med 6(7): e1000097. doi:10.1371/journal.pmed1000097

For more information, visit: **www.prisma-statement.org**.

For peer review only - http://bmjopen.bmj.com/site/about/guidelines.xhtml

BMJ Open

The role of active patient involvement in undergraduate medical education: A systematic review

Journal:	*BMJ Open*
Manuscript ID	bmjopen-2020-037217.R1
Article Type:	Original research
Date Submitted by the Author:	14-Apr-2020
Complete List of Authors:	Dijk, Stijntje; Erasmus MC<br>Duijzer, Edwin; University Medical Centre Groningen<br>Wienold, Matthias; Wissenschaftliche Dienste und Projectbaratung
**Primary Subject Heading**:	Medical education and training
Secondary Subject Heading:	Medical education and training
Keywords:	EDUCATION & TRAINING (see Medical Education & Training), MEDICAL EDUCATION & TRAINING, HEALTH SERVICES ADMINISTRATION & MANAGEMENT

SCHOLARONE™
Manuscripts

I, the Submitting Author has the right to grant and does grant on behalf of all authors of the Work (as defined in the below author licence), an exclusive licence and/or a non-exclusive licence for contributions from authors who are: i) UK Crown employees; ii) where BMJ has agreed a CC-BY licence shall apply, and/or iii) in accordance with the terms applicable for US Federal Government officers or employees acting as part of their official duties; on a worldwide, perpetual, irrevocable, royalty-free basis to BMJ Publishing Group Ltd ("BMJ") its licensees and where the relevant Journal is co-owned by BMJ to the co-owners of the Journal, to publish the Work in this journal and any other BMJ products and to exploit all rights, as set out in our licence.

The Submitting Author accepts and understands that any supply made under these terms is made by BMJ to the Submitting Author unless you are acting as an employee on behalf of your employer or a postgraduate student of an affiliated institution which is paying any applicable article publishing charge ("APC") for Open Access articles. Where the Submitting Author wishes to make the Work available on an Open Access basis (and intends to pay the relevant APC), the terms of reuse of such Open Access shall be governed by a Creative Commons licence – details of these licences and which Creative Commons licence will apply to this Work are set out in our licence referred to above.

Other than as permitted in any relevant BMJ Author's Self Archiving Policies, I confirm this Work has not been accepted for publication elsewhere, is not being considered for publication elsewhere and does not duplicate material already published. I confirm all authors consent to publication of this Work and authorise the granting of this licence.

For peer review only

Title: The role of active patient involvement in undergraduate medical education: A systematic review

Stijntje W. Dijk[1], Edwin Duijzer[2], Matthias Wienold[3]

[1] Medical student, Erasmus University Medical Center, Doctor Molewaterplein 40, 3015 GD Rotterdam
[2] Medical doctor, University Medical Center Groningen, Hanzeplein 1, 9713 GZ Groningen
[3] Patient advocate, Wissenschaftliche Dienste, Rigaer Straße 30, 10247 Berlin

Corresponding author:
Stijntje W. Dijk, Erasmus University Medical Center, Doctor Molewaterplein 40, 3015 GD Rotterdam
email address: 340974sd@student.eur.nl

For peer review only

ABSTRACT

OBJECTIVES: To identify the scope of active patient involvement in medical education, addressing the current knowledge gaps relating to rationale and motivation for involvement, recruitment and preparation, roles, learning outcomes, and key procedural contributors.

METHODS: The authors performed a systematic search of the Pubmed database of publications between 2003 and 2018. Original studies in which patients take on active roles in the development, delivery or evaluation of undergraduate medical education and written in English were eligible for inclusion. Included studies references were searched for additional articles. Quality of papers was assessed using the MMAT tool.

RESULTS: 49 articles were included in the review. Drivers for patient involvement included policy requirements and patients own motivations to contribute to society and learning. Patients were engaged in a variety of educational settings in and outside of the hospital. The vast majority of studies describe patients taking on the role of a patient-teacher and formative assessor. More recent studies suggest that patients are increasingly involved in course and curriculum development, student selection and summative assessment. The new body of empirical evidence shows the wide range of learning objectives were pursued through patient participation, including competencies as professional, communicator, collaborator, leader and health advocate, but not scholar. Measures to support sustainable patient involvement included longitudinal institutional incorporation, patient recruitment and/or training, resource support and clear commitment by faculty. The importance and advantages of patient involvement were highlighted by students, faculty and patients themselves, however, organizations must continue to consider, monitor and take steps to mitigate any potential harms to patients and students.

DISCUSSION: This systematic review provides new knowledge and practical insights to physicians and faculty on how to incorporate active patient involvement in their institutions and daily practice, and provides suggested action points to patient organizations wishing to engage in medical education.

OTHER: No funding was obtained for the performance of this review.

Article Summary

Strengths and limitations

- This systematic review is the first of its kind focused specifically on undergraduate medical education, providing practical guidance to educators, students and patients with ambition to improve work in healthcare professionals education.
- The study provides novel insights in the wide range of learning objectives pursued through patient participation, the educational settings and roles in which patients participate, and practical support systems that enable patient engagement.
- As many articles written by patients on their experiences in involvement in medical education may only be found in grey literature, including blog posts, conference statements and patient organization newsletters, this review may have missed their viewpoints.

For peer review only - http://bmjopen.bmj.com/site/about/guidelines.xhtml

INTRODUCTION AND RATIONALE

In recent decades, the involvement of patients in medical education has been advocated for increasingly and has become common practice adopted by reformers of medical education. (1) Patients and their narratives are no longer just used as subjects for "learning material" in clinical training. Towle et al. identified different levels of involvement, from paper based involvement to involvement at the institutional level as co designers of the medical curriculum in addition to sustained involvement as patient-teachers in education, evaluation and curriculum development. (1-4) Medical educators are now seeing the value of linking medical students with patients and their families and communities to foster awareness of the importance of longitudinal relationships, to improve students social interaction skills and to facilitate learning of coping with illness in the real world. (5, 6)

Despite an increasingly collaborative role of patients in medical education, there is much to be learnt about how to embed it, and how to develop systematic, institution-wide approaches to planning patient involvement in all levels of medical education. (3, 7) The drive towards a more equal partnership in clinical decision making and patient-centered care, fueled by national and international guidelines, promote the expansion of the efforts towards developing a culture where partnership in medical education becomes the norm. (3, 5-15)

As researchers have used varying definitions of active patient involvement in medical education, they have used varying inclusion criteria in their literature searches. This has resulted in overlap of included papers, and limited the generation of a common theoretical framework and terminology. (3, 16) Previous studies have identified major gaps in the knowledge base relating to short and long term learning outcomes, ethical issues, psychological impact and key procedural contributors like recruitment, selection and preparation. There is also limited information of the cost-effectiveness of active patient involvement.
Since the publication of the last systematic reviews (1, 17-19) and non-systematic reviews (2, 8, 20) of patient involvement in medical education, many new studies have been published. Previous reviews addressed only the patient teacher role (1), teaching and assessing one specific skill (intimate examination) (17), included simulated patients (17), included post-graduate medical education (18) or addressed all health care professions. (2, 17, 20) A recent systematic review provided a comprehensive overview of the involvement, outcome and reason behind involvement mainly from learner perspective. (19)
Our paper reviews and summarizes the most recent literature using a broad definition of patient involvement consisting of any form of involvement that is beyond merely incidental passive involvement, in any field or setting of undergraduate medical education. By adopting this definition we are able to extend the scope and amount of research data in order to increase the practical knowledge base on active patient involvement and in order to give ground to an improved theoretical framework and common terminology. Our study takes a novel approach by focusing primarily on the patients' perspective on their involvement.

METHODS

Design

Our literature search employed a systematic review method looking for active patient involvement in medical education defined as the direct, involvement of real patients and community members in the development, delivery or evaluation of undergraduate education of medical students.

Search Strategy

The authors performed a search through PubMed on July 12th 2018. The search terms used on their own and in combination included*: patient*, communit*, involvement, engag*, cooperat*, collaborat*, represent*, medical education, curriculum, medical student**. Search criteria were reviewed by a hospital information specialist. The full search strategy can be found in **[SUPPLEMENTARY FILE 1]**

All articles published in English between January 1st 2003 and July 12th 2018 and reporting primary empirical research that addressed the active participation of patients in undergraduate medical education were eligible for inclusion. Studies with

For peer review only - http://bmjopen.bmj.com/site/about/guidelines.xhtml

simulated patients or actors, patients solely undergoing examinations or patients who were only being observed in wards were excluded from the review.

We assessed articles based on title and abstract in the first round, and in a second round based on full text. References of all included articles were analyzed for additional studies that matched the original inclusion criteria. All reviews that complied with the inclusion criteria were additionally assessed for relevant references. Only original research articles were included in the final analysis.

Quality assessment of included studies

As our review included papers of qualitative, quantitative, and mixed-method designs, two authors (SD and ED) applied the Mixed Methods critical appraisal Tool (MMAT) to assess the methodological quality of studies**[SUPPLEMENTARY FILE 2]**.(21) Studies were not excluded based on assessed methodological quality.

Data extraction and synthesis

All authors (SD, ED, MW) used a pre-piloted standardized form to extract data from included studies. A second author checked the extracted data were accurate, and discrepancies were resolved through discussion. The following data were recorded: authors, year of publication, journal, country intervention, study type, abstract, study setting, financial aspects reported, number of patients in intervention, number of students in intervention, patient characteristics, patient motivations to join, recruitment practices, training and preparation practices, role of patient organizations, type of patient involvement, outcome measurement, organizational remarks on sustainability and pursued learning outcomes. To explore this range of learning outcomes, we categorized intended learning outcomes according to the CanMEDS™ framework as a commonly applied competency framework within medical schools. (22) We organized extracted data in related themes to explore connections and discrepancies between data elements. We opted not to use any of the existing frameworks for grouping potential roles patients take on. In the discussion section, we compare our findings of the diversity of roles with the existing taxonomy by Towle et al. (2)

RESULTS

Study selection

The initial search resulted in 769 articles, of which 95 were selected for further review based on the title and/or abstract. These 95 articles were independently read by two authors (SD and ED) and included based on the specified criteria. Consensus between reviewers was 91,2%. The remaining articles were included based on consensus after a short discussion. Main reasons for exclusion where wrong article type (conference abstracts or commentaries) and studies that did not concern active participation of patients. The review of references resulted in 11 additional articles for inclusion. The characteristics of the 49 studies that met inclusion criteria are presented in [**FIGURE 1, SUPPLEMENTARY FILE 3**].

We used the extracted from included studies to synthesize the evidence in the following subsections:

- Rationale for involving patients in medical education
- Patient recruitment and selection
- Patients' preparation to participate in medical education
- Roles patients take on in medical education
- Learning objectives pursued through patient involvement
- Concerns about the involvement of patients
- Patients' views on the impact of their involvement
- Financial implications of patient involvement
- Roles of patient organizations
- Measures to ensure the sustainability of patient involvement

Rationale for involving patients in medical education

Several authors referred to government policy mandating patient participation in medical education, namely the UK Department of Health and the UK General Medical Council (16, 23, 24), the Australian Medical Council (25) and the World Health Organization (WHO) (5) as a rationale for their patient involvement initiatives. Besides these political drivers, cited rationales were: to teach students patient-centered and interprofessional care (24, 26-28); to introduce students to chronic illness care (29-31); to create a multicultural learning environment; to practice social accountability and an inclusion agenda (32-37); to make education more

engaging, powerful, and transformative (38); and to empower patients. (39) Patients mentioned that they felt a sense of responsibility to the broader community in shaping the future health workforce (24, 32) and improving the healthcare system. (24, 33, 40)

Patient recruitment and selection

The most frequently reported methods to recruit patients were through existing university partnerships and existing programmes (28, 33, 41-46), advertisements through press or social media or posted in health facilities (26, 34-36, 47, 48), through community- and patient organizations, through personal connections and previous participants (32, 38, 49-51), and through health professionals (52-55). In one project, where students shadowed a patient with a chronic condition, students were asked to recruit patients themselves. (30)

Selection criteria were generally set up broadly, inviting any patient or community member. General criteria for patient educators included good communication skills, affinity for teaching, aptitude for further learning, enthusiasm, time to commit to the study, as well as being fully mobile and being able to cope with repeated physical examinations. (48, 54, 56) In some cases patients were required to have representative physical signs of their disease. (53, 54, 56, 57)

Patients' preparations to participate in medical education

20 articles mention preparation of patient teachers. The duration of the preparation ranged from substantial training sessions of 100 hours in total (42) to the majority of programmes providing solely written information or a single orientation session of 1 to 1.5 hours (24, 30, 32, 39, 43, 49, 52, 57). Training programmes for patients in teaching musculoskeletal skills were the most extensive and were delivered by medical or educational experts. (42, 47, 54, 56, 58, 59) Other preparatory sessions were less formal and were facilitated by faculty educational experts, students or peers.

The primary aims of the preparatory programmes varied. One study underscored the importance of patient educators being aware of the course goals in order to safeguard student learning outcomes. (53) Others mentioned aiming to serve the needs of patients in building their confidence, providing skills training (57) and providing knowledge related to the educational process. (23, 39, 48, 60) These sessions addressed approaches such as problem based learning, how to deliver a presentation, co-facilitation methods, and how to provide effective feedback. Preparation also provided opportunities for anticipating benefits and challenges such as conflicts, emotions, unmet expectations, using methods of coaching, supervision and debriefing. (40, 61)

One article mentions finishing the preparation of patient educators with a short quiz as an assessment tool and having a graduation session before starting to teach. (28) Another article describes the use of a post-training satisfaction questionnaire to help ensure that patient educators felt ready to teach. (47) The majority of papers did not address assessing patients prior to them taking on their roles.

Towle et al. (2013) discussed the tension between preparedness of patient educators and authenticity of education in both form and content. (49) They highlight the critical role of the community organization representatives who can be brokers between the two cultures of academia and community. One article describes an intervention in which the patient educators explicitly have not received training, so the student-patient encounters would be as authentic as possible. (53) Another article described that the collaboration between patients and educators allow for mutual learning without an authentic patient perspective being lost. (39)

Roles patients take on in medical education

The main categories of roles that we identified are divided in the areas of a patient as a teacher, an assessor, a curriculum developer and a student selector. [**BOX 1**]

Box 1: Identified patient roles in medical education

Role	Specification
**Patient Teacher**	Deliver clinical skills sessions on history taking, counseling and physical examination<br>Deliver formative feedback during teaching sessions<br>Share experiences in healthcare or personal aspects of their lives in teaching sessions, small group sessions, individual mentorship and coaching, or through the creation of videos

**Patient Assessor**	Deliver formative feedback during teaching sessions<br>Perform summative assessments during OSCE's
**Patient Curriculum developer**	Evaluate the medical program<br>Act as a curriculum steering committee member<br>Were consulted to provide recommendations on disease specific courses through focus groups, world cafés or discussions with community leaders<br>Participate in development of courses related to their illness or social conditions, or overarching competencies and community based learning<br>Develop courses delivered by patient teachers autonomously<br>Identify end-competencies for graduates<br>Identify desired curriculum characteristics<br>Be consulted on the strategic development of a new medical school department
**Patient selection committee member**	Participate in the selection of students applying for the medical program<br>Assess candidates' communication skills, sensitivity, compassion and empathy towards societal contexts and needs.

1. Patient as teacher

The role of a teacher was cited most frequently. Patient teachers gave clinical skills practicals on history taking and physical examination sessions on their own condition such as musculoskeletal disorders. (42, 47, 54, 56, 58, 59, 62) They were trained to teach students skills and deliver immediate feedback, which stimulated further learning. (56)

Several groups of patients with disabilities acting as patient teachers with disabilities gave practicals on communication skills and history taking. (48, 53, 57) People living with HIV participated as teachers during a simulated clinical encounter in which students provided counseling. (60)

In addition to clinical skills, patients taught students about their experiences of overall management of care, and the personal aspects of their lives. These ranged between practical physical and home adjustments, to psychological, social and behavioural issues impacting them and their family. Teaching was done through panel discussions and small group sessions (24, 43, 63) as well as visits to the community and patients' homes. (31, 52) Patient teachers with chronic conditions acted as mentors, and met regularly with students. (27, 29) Patients taught students patient-centeredness and interprofessionalism (23, 24, 31, 38, 49), community-centeredness, cultural competence and ethics. (33, 64) Patients could choose their own teaching method, such as telling their stories and stimulating reflection. (38) A group of patients living with intellectual and developmental disabilities also participated in the creation of learning materials, through videos sharing their perspectives and stories. (65)

Most patients in the study by Jackson et al. (2003) considered themselves not as teaching, but having a role of partnership, explanation and sharing certain aspects of their illness. (52)

2. Patient as assessor of students' competence

In addition to formative student assessment, such as feedback during teaching sessions, patients participated in high stakes summative assessments, such as the final year Objective Structured Clinical Examination (OSCE). (39) Patients also provided written feedback to student essays, which were used for formal assessment. (39) Patients assessed mostly non-cognitive domains of student performance. (35, 46) Medical educators interviewed by Jha et al. (2009) believed there was a role for patients in assessing whether students made them feel at ease and whether students asked the right questions. (66) Whilst patients and medical educators in the study by Raj et al (2006) praised patients' assessments, students expressed their concerns whether patients could reliably assess clinical skills or whether they were likely to be too lenient. (54)

3. Patient role in curriculum development

Community members were motivated to participate in curriculum development. (35) While they were not seen as medical experts, they did have an interest in ensuring optimal health care for themselves and their families. (26)

Community members actively participated in the planning, implementation, and evaluation of the educational program. (46, 49) Patient-teachers had autonomy from the stage of planning to the stage of delivery of teaching. (43, 67) Several patients were members of the steering committee for the psychiatry curriculum (28) and the interprofessional education curriculum. (49)

Patients were involved in the development of courses related to their illness or social conditions. (35) Aboriginal delegates provided recommendations for the development of an Aboriginal health curriculum and community placement. (33, 44) Focus group meetings with Native Hawaiians were held to define a cultural competencies and health disparities curriculum. (37) A world

For peer review only

café discussion was similarly held to inform the curriculum on transgender health. (36) Minority community members provided input on curricular design, especially on the content of the cancer disparities curriculum. (35)

Beyond the disease- or competency specific courses, patients were involved in consultative meetings with stakeholders to identify desirable attributes, competencies of graduates, and development of a community based learning environment. (45) Patients were also consulted on the desired characteristics of the curriculum. (68) One medical school sought input for the strategic development of the department of population health in a new medical school through focus groups. (69)

4. Patient role in selection of students to medical schools

Community members were invited to join a panel together with clinicians and academic staff members to select students applying for the Graduate Entry Medical Program. (32) Members of the community were invited to be a part of the student selection process and team, especially in assessing candidates' communication skills as well as sensitivity, compassion and empathy towards social contexts and societal needs. (45)

Collaboration between faculty and patients

The role of faculty members in the collaboration with patient teachers varied. Some patient teachers worked under the supervision of a clinical preceptor. (60) In other sessions, patients were co-facilitators with practitioners. (23) Workshops were led by patient teachers and facilitated, but not controlled by faculty. The faculty member's role was to support the direct learning between students and mentors. (49) Faculty was not always present in meetings but could provide background support, such as setting broad topics for discussions. (46) Patient-teachers stated that program support was essential for participation, allowing them to transform from teaching individual messages to teaching universal lessons. (43)

Lay participants of one study regarded sharing of curriculum ownership as necessary to acknowledge the importance of lay perspectives, whereas faculty participants presumed ownership of curriculum development. (26) Faculty in the study by Jha et al. were not clear on how to involve patients more fully in assessments or course development, nor were they convinced of the appropriateness of doing so. Some faculty members expressed their experiences of working with patient-assessors and course developers as tokenistic. (66)

Learning objectives pursued through active patient involvement

Learning outcomes of patient participation were quantitatively assessed on the subject of musculoskeletal (MSK) examination skills in four randomized controlled experiments (42, 47, 54, 62) and two further studies. (56, 59) No difference was observed in increased structured clinical examination (OSCE) scores when comparing sessions delivered by trained patient educators with sessions delivered by rheumatology staff together with a passive patient undergoing examination (54, 62) and sessions with a non-MSK specialist physician (42). In the experiment by Humphrey et al., significantly fewer faculty-taught students failed (0 out of 32) than patient educator-taught students (5 out of 30). (62) Students rated faculty educators higher than patient educators (4.13 versus 3.58 on a 5-point Likert scale). (62)

When students were taught by a patient teacher in addition to the regular faculty-led sessions, their OSCE scores increased more compared with students participating in the regular curriculum. (47) An intervention by de Boer et al. (2011) offered students the opportunity to participate in two non-obligatory real patient learning sessions in the preclinical musculoskeletal disorders block. (59) Students who participated scored significantly higher at the end-of-block test.

Oswald et al. (2011) examined how teaching was different between patient educators and physician educators when teaching MSK physical examination skills. (58) Video recordings show that trained patient educators were more consistent in content and style by consistently covering all major joints. Bokken et al. (2010) assessed student's perspectives on instructiveness of real patients versus simulated patients. (53) Overall instructiveness was marked high. Students regarded real patients as more authentic and the encounters more useful in practicing physical examination

In the intervention study by Jaworksy et al (2017) medical students provided HIV test counseling to patient instructors. (60) Pre- and post-intervention scores of the validated Health Care Provider HIV/AIDS Stigma Scale (70) demonstrated a significant decrease (68.74 versus 61.81). Students reported increased comfort in providing HIV-related care (10.24 versus 18.06). Similarly, students in intervention studies with patient teachers living with physical or mental disabilities, demonstrated an improved attitude

(28), increased levels of comfort in communication (57), increased levels of self-efficacy and confidence (63, 65) and higher mean performance scores across all interview stations when compared to a control group. (65)

Students in the study by Rees et al. described the encounters with patients as more motivating compared to textbook learning. (71)

Wide ranges of learning outcomes of education with patient participation were mentioned in the qualitative studies identified by this review. To explore this range of outcomes a categorization is used according to the CanMEDS™ framework, developed by the Royal College of Physicians and Surgeons of Canada. (22) [**BOX 2**]

Box 2: Aspired learning objectives for medical students based on the CANMEDS™ framework

CANMEDS role	Specification
**Communicator**	Apply a patient-centered approach to interviewing and care<br>Adopt to the unique needs and preferences of each patient as an individual, recognizing their needs.<br>Communicate using a patient-centered approach that encourages patient trust and autonomy, recognizing their expertise in care and seeing them as part of a team.<br>Create an environment for patient comfort, dignity, privacy, engagement and safety by using a non-patronizing and nonjudgmental attitude, recognizing biases.<br>Apply communication skills to share information and explanations that are clear and accurate, checking for understanding, using communication skills that help patients make informed decisions
**Collaborator**	Work effectively with physicians and colleagues in the health care professions through interdisciplinary teams
**Leader**	Contribute to the improvement of health care delivery through understanding the broader health care system, and how it affects patients.
**Professional**	Demonstrate a commitment to patients and society, by applying best practices and adhering high ethical standards, dealing with ethical complexity of clinical practice.<br>Demonstrating a commitment to the profession, reflecting on role models and professional identity, keeping fellow physicians and oneself to high professional standards, and understanding patient views on clinical errors<br>Demonstrating commitment to physician health and well-being by learning to cope with uncertainties, emotions and stress<br>Exhibit appropriate professional behaviors and relationships, demonstrating respect for diversity, respect and compassion.
**Health Advocate**	Responding to individual patient's health needs by advocating with the patient within and beyond the clinical environment, specifically for patients in vulnerable situations.<br>Awareness of the importance of physician and patient advocacy<br>Working with communities or patients to identify determinants of health that affect them
**Scholar**	No paper explicitly described the aim of developing the role of scholar
**Medical Expert**	Performing patient-centered clinical assessments and establishing a management plans<br>Establishing plans for ongoing, continuity of care.<br>Understanding the complexity of practicing medicine<br>Integration of theory into practice

Communicator

Several authors mentioned patient-centered care as the main outcome of education involving patients. (23, 27, 29, 49, 67) Patient-centeredness included the ability to see patient-mentors as individuals (27), the importance of patient autonomy and expertise in care (64), adopting a non-patronizing and nonjudgmental attitude (55), recognizing patients' needs (41) and seeing the patient as a capable part of the team. (41) Jha et al. pointed out that active patient involvement by itself demonstrates an equal partnership (66) and Rees et al. concluded that this approach helps students to develop a holistic perspective of health care. (71) More generally, patient participation was associated with increased understanding of the importance of communication (27, 29), building and improving communication skills (55, 71), empathy, listening skills and respect. (71)

Collaborator

McKinley described an education programme in which students undertake a home visit to a patient with a chronic condition (31), where students demonstrated increased understanding of interdisciplinary teams in management of chronic conditions in their reflective assays. Four authors described interprofessional education programmes in which patients are involved. (23, 27, 44, 49)

For peer review only - http://bmjopen.bmj.com/site/about/guidelines.xhtml

Leader

In a longitudinal mentor program with medical, physical therapy, occupational therapy, nursing, and pharmacy students teaming up with a patient-mentor students reported a deeper understanding of the healthcare system. (27). A yearlong student mentor program gave students an experience in and appreciation of continuity of care. (55)

Professional

Various qualitative studies suggested that patient involvement can attribute to dealing with ethical complexity in clinical practice and patients' perspectives on clinician error (64) and developing reflective skills. (29, 48, 55) Reflecting on role models some authors referred to broadening understanding of the role of the health care provider (27), qualities of remarkable clinicians that inform personal ideals (64), creating a future professional model (55) and professional identity. (71) Experiences with real patient-educator encounters could also help in coping with uncertainties, emotions and stress. (71)

Exposure to patient-educators from within specific patient or minority groups helped students to increase positive attitude toward chronic conditions and elderly (27, 31), patients with mental health problems (29) or disabilities. (48)

Health advocate

Students reflected on the importance of patient advocacy in day-to-day practice in a study on experiences within an ethics and professionalism module with patient-mentors. (64) More specifically, students were empowered to advocate for patients when they are in vulnerable situations. One of the aims of the education program described by Saketkoo et al. was to develop an awareness of the impact of physician advocacy, specifically in the context of people with disabilities. (63) A pre- and post-test showed that this awareness increased significantly with the programme.

Scholar

No programmes have explicitly described the aim of developing the competency of scholar.

Medical expert

The role of medical expert integrates all other roles by applying medical knowledge, clinical skills, and professional values in the provision of high-quality and safe patient-centered care. Two articles mentioned that patient participation supports students' learning, by recognizing the complexity of practicing medicine. (41, 48) Jha et al described the patient as providing an illustration of the theory in practice, thus enhancing students' understanding and recall. (66)

Concerns about the involvement of patients

Various authors have also raised concerns when involving patients as teachers in medical education. Some faculty educators were concerned that patient stories might be so traumatic that students would require support or debriefings to deal with the resulting emotions. (66, 71) In a qualitative study, students felt "pressured" by service users asking them for information and advice, rather than asking their clinicians, or when service users divulged information to students that they had not told their clinicians. (71) Students worried about giving incorrect information to patients.

Students expressed reservations that they were only getting the view of one person, which could lead to a biased perspective. (23) Students were also concerned that patients might have difficulty discriminating between poor and good performance, and are likely to be too lenient in feedback or assessment. (54) Students in the study by Henriksen and Ringsted (2014) expressed skepticism about patients' knowledge (67) and expressed concerns about unstructured experiential learning in a context where patients had autonomy in both planning and delivering the teaching encounter. (67) In a different study, staff members expressed the concern that the impact of the patient experience might be reduced if the same patient was involved in the same programme too often. (66)

Patients' views of the impact of their involvement

Patients described a strong sense of having a meaningful contribution and personal fulfillment, because they were teaching patient centeredness (24, 72), offering their body and authenticity, bolstering students' confidence, (72) fulfilling their responsibility to the broader community (24, 32) and improving the health care system. (24, 33, 40)

On an individual level patients described material, professional, personal and emotional benefits. Material benefits included receiving tangible rewards such as gifts (43) and receiving a full medical checkup. (40) Patient educators with back pain involved

For peer review only - http://bmjopen.bmj.com/site/about/guidelines.xhtml

in teaching medical students stated their participation improved the management of their own back pain, and improved confidence in voicing their needs in consultations with physicians. (47) Some patients felt that they received more time and attention from their healthcare professionals when they were teaching. (71)

Patients described professional growth and personal fulfillment from being involved in the selection process of students. (32) Hatem et al. reported practical benefits for patients including getting better at finding health care providers and increased knowledge of their medical condition. (43)

The drawbacks and risks associated with patient involvement in medical education included being confronted with stigmatizing assumptions, vulnerability of self-disclosure and spontaneous questions-answer exchanges. A patient educator teaching on the subject of HIV for example described the experience of being very frustrated with one man's lack of knowledge and ignorance about the disease. Patients also drew attention to the fact that unanticipated disease progression had an impact on their ability to teach. In some cases this resulted in them pulling out of their commitment as teachers. inevitable loss among patient educators. (43) Patients also described a sense of vulnerability to negative and non-appreciative reactions from students. (40, 47) Initially, mentors were commonly anxious and unsure about whether what they shared was of benefit to students. (29) Half of the patients involved in the community based intervention in a socio-economic deprived area expressed feelings of anxiety, apprehension or nervousness prior to the interview, although in all cases patients felt that this was normal. (52) In addition to the word ''vulnerable'', patients employed terms like ''exposed'', ''frightened'', ''tired'', ''stressed'' and ''harrowing'' to emphasize service users' feelings within the clinical education environment. (71) Some even described it as traumatic for mental health service users to repeatedly tell their stories. (71)

Financial implications of patient involvement in medical education

Nineteen articles commented on any financial aspect of the interventions, ranging from reimbursement of patients' expenses, payments of honoraria, organizational costs, or perceptions of cost. Economic and financial resources, however, have not been explored in a way that they can be systematically compared. Reported financial costs included $800 for a disability skills workshop (63), £800 for a physical examination training (54), and £2640 for the overall Patient Partners programme (47). Ten studies offered participating patients remuneration through honoraria between €8 per hour and £350 per day (43, 53, 54) or an unspecified amount. (28, 35, 39, 48, 50, 57) Four studies offered reimbursement of patient expenses such as travel, phone or mail costs. (24, 34, 47, 65) Some staff feared that cash patients needed to spend on refreshments or public transport would be a barrier for their participation. (73)

Medical educators suggested that patient involvement was a costly endeavor, both in financial investment as well as staff time. (71) Only one article commented on cost-effectiveness, noting that patient-led teaching is a cost-effective method compared to physician-led teaching, but did not provide an economic evaluation. (54) No paper provided a cost-effectiveness or cost-benefit analysis.

Roles of patient organizations

The most cited interaction between patient organizations and medical faculties was the use of patient organizations and their networks for the recruitment of individual patients as community members or members of condition-specific support groups. (24, 28, 29, 36, 49, 55, 56, 60, 69) In the paper by Baral (2016), representatives from rural communities and consumer groups were consulted by the Medical School Steering Committee for the development of the Academy of Health Sciences curriculum. (45) Representatives of community-based patient advocacy and support organizations took part in the advisory group of the intervention in the study by Towle et al (2013). (49) Not all of these representatives of patient organizations were patients themselves. They were described as brokers between two cultures of academia and community.

The University of Leeds worked with a dedicated internal patient group named "The Patient Voice Group", consisting of lay people who use their experiences to inform their roles as teachers and researchers. This group was involved in formative and summative assessment. Additionally, a Patient and Public Involvement Manager who provided ongoing support was assigned within the school. (39)

Some medical educators made the explicit choice not to collaborate with patient organizations, due to a fear of working with politicized groups. (71) They did make a decision to include patient groups to allow multiple voices to be heard in order to prevent criticisms of tokenism. Patients stated that participating in groups gave them support and companionship from their peers. (71)

For peer review only - http://bmjopen.bmj.com/site/about/guidelines.xhtml

Patient organizations wishing to engage in medical education may wish to consider some of the practical points as described in [**BOX 3]**

Measures to ensure the sustainability of patient involvement

The key factors identified in sustaining patient involvement were the provision of adequate resource support, formal acknowledgement of the value of lay contributions, and a clear faculty commitment to change following lay input. (26)

Institution-wide incorporation of social accountability or patient-centered education and medicine in the university's mission and vision statement or strategic plan were cited in several papers to ensure patient and community involvement. Authors emphasized that the resource intensity of patient involvement program requires the university to value its patient-centered underpinnings in order to be sustainable. (26, 44, 45, 61) The incorporation of initiatives as ongoing modules in the curriculum achieved sustainable patient involvement rather than sporadic involvement. (53, 63)

In the university of Leeds, a permanent patient voice group was incorporated in the institution. (39) The institution appointed a Patient and Public Involvement Manager to provide ongoing support. Some initiatives chose to work in partnership with existing institutions, implemented at a school-wide level (45) or focused on one condition such as arthritis. (58) Gaver et al. (2005) identified the process of establishing commitment among volunteering organizations and families as a key challenge to the sustainability of patient involvement. (55)

Medical educators commented that if patient-educators were paid and seen as an employee of the medical school, they might take on the role more seriously and become more reliable, as well as being seen as a respected part of the educational team. (66)

DISCUSSION

This review systematically evaluated 49 primary empirical studies and was aimed at providing updated integrated evidence on the role and impact of the active involvement of patients in medical undergraduate education. The new body of empirical evidence shows the increasing range of learning objectives and educational settings in which patients play an active part in undergraduate medical education.

Our study found that patients described material, professional, personal and emotional benefits of participating in medical education. In addition to expected benefits, several authors mentioned policy mandates as rationale for initializing patient involvement programmes. Several studies however reported on the potential harms and negative experiences, such as fear of stigmatization, tokenism, or lacking structure of teaching session. Concerns related to patient involvement coming from students, faculty and patients themselves should remain closely monitored in a systematic manner and addressed appropriately.

Included papers described various types of roles for patients, but the vast majority of papers cited the role of a patient teacher, similarly to previous reviews. (1, 17-19) More recent papers suggest that patients are increasingly involved in curriculum development. Most of these initiatives were incidental and were lacking institutional incorporation and longitudinal involvement.

The patients' roles identified in this review are largely in accordance with level 3-6 of the spectrum proposed in the review by Towle et al. (2010). (2) [**BOX 4**] We additionally identified new roles that could not be ascribed to one specific level on this spectrum. In one role, patients did take on roles as equal in curriculum development, but only to specific courses rather than the curriculum as a whole, falling between Towle's levels 4 and 5. In another role, patients were consulted in institution level topics and curriculum development beyond specific courses, but rather than being equal partners, they were consulted in a faculty driven initiative, displaying partial elements of Towle's level 4, 5 and 6. (2)

The learning objectives identified in this review encompassed all but one of the CanMEDS™ roles for future physicians. This demonstrates that patient's involvement is continuing to gain a larger influence on a diverse range of aspects of the medical curriculum.

Measures to support sustainable patient involvement included longitudinal institutional incorporation, patient recruitment and/or training, resource support and clear commitment by faculty. The importance and advantages of patient involvement were highlighted by students, faculty and patients themselves, however, organizations must continue to consider, monitor and take steps to mitigate any potential harms to patients and students. Only few papers reported on the financial aspects related to patient involvement, which should be further investigated to help support feasibility

For peer review only - http://bmjopen.bmj.com/site/about/guidelines.xhtml

An important limitation was the lack of common terminology in the existing literature, potentiating the risk of missing relevant articles, which has been previously reported as a limitation by other review authors. (2)

Our systematic review included only original literature from peer-reviewed journals. As many articles written by patients on their involvement in medical education may only be found in grey literature, including blog posts, conference statements, and patient organization newsletters, this review runs a risk of having missed important aspects of patient views on this topic. Additionally, only studies written in English were included, which may have led to bias in selected papers. The majority of included papers were from North-America (N=23), Europe (N= 17) and Australia and New Zealand (N=7).

The majority of included studies was qualitative (N=38), others were mixed methods (N=5) or qualitative (N=6). Only few of the included papers used control groups (N=7). In most cases, students and patients participated in interventions on a voluntary basis, which may limit the generalizability of findings to the wider population. Another important factor that may have introduced bias, is that most studies were not (possible to be) anonymized, or were part of student assessments, which introduces a risk of responses being subject to social desirability bias.

Future research should focus on the long-term effects for patients, students and the healthcare system, especially on the subjects of patient-centeredness and shared decision making. This gap in research limits recommendations that can be made based on current literature. Additionally, no paper performed an economic evaluation of patient involvement, which may be a critical factor for decision makers in educational policy. Finally, more research is needed to update existing frameworks for patient involvement to the newly identified roles and needs patients have in medical education.

CONCLUSION

It has been over 40 years since the first article on patient involvement in medical education was published. Today both the medical education community and the patient community have joined together in the movement to promote patient-centeredness. This systematic review provides knowledge and practical considerations that can aid curriculum developers that wish to sustainably incorporate active patient involvement in their institutions, and patient organizations wishing to engage in medical education.

Contributors

SD and ED jointly developed the review protocol and search strategy, and SD, ED and MW jointly developed the data extraction sheet. The search strategy was critically reviewed by a hospital information specialist. Data collection was carried out by SD and ED and extraction was carried out by SD, ED and MW. The paper was written jointly by all three authors.

Acknowledgements

The authors would like to thank Tessa Richards (BMJ Patient and Public Involvement lead) for her valuable comments and suggestions.

Data sharing

The authors confirm that the data supporting the findings of this study are available within its supplementary materials.

Funding

No funding was obtained for the performance of this review.

Competing interests

We have read and understood BMJ policy on declaration of interests and declare that we have no competing interests.

Ethical approval

Ethical approval for this study was not applicable, as no human or animal subjects were involved.

Patient and public involvement statement

The initial impulse for the research initiative came was formed following a collaboration between the authors who were representatives of the International Federation of Medical Students' Associations (IFMSA) and the International Alliance of Patient Organizations (IAPO). MW, co-author, was involved in all stages of data analysis, discussion and writing of the paper. The initial

For peer review only - http://bmjopen.bmj.com/site/about/guidelines.xhtml

draft was presented and discussed at the European Patient Forum 2019 in a plenary session with over 300 patient representatives present. The authors incorporated the feedback from the European Patient Forum and BMJ's PPI lead**,** Tessa Richards, into the final draft. The results will be disseminated through the networks of IAPO to reach patient organizations, and will be presented at future patient and medical education conferences.

License

The corresponding author has the right to grant on behalf of all authors and does grant on behalf of all authors, a worldwide licence to the publishers and its licensees in perpetuity, in all forms, formats and media (whether known now or created in the future), to i) publish, reproduce, distribute, display and store the Contribution, ii) translate the Contribution into other languages, create adaptations, reprints, include within collections and create summaries, extracts and/or, abstracts of the Contribution, iii) create any other derivative work(s) based on the Contribution, iv) to exploit all subsidiary rights in the Contribution, v) the inclusion of electronic links from the Contribution to third party material where-ever it may be located; and, vi) licence any third party to do any or all of the above.

Transparency declaration

The lead author affirms that this manuscript is an honest, accurate and transparent account of the study being reported; that no important aspects of the study have been omitted; and that any discrepancies from the study as planned (and, if relevant, registered) have been explained

References

1. Wykurz G, Kelly D. Developing the role of patients as teachers: literature review. BMj. 2002;325(7368):818-21.
2. Towle A, Bainbridge L, Godolphin W, Katz A, Kline C, Lown B, et al. Active patient involvement in the education of health professionals. Medical education. 2010;44(1):64-74.
3. **GMC**. Patient and public involvement in undergraduate medical education. Advice supplementary to Tomorrow's Doctors (2009). General Medical Council. Great Britain: GMC; 2011.
4. Dijk SW. Meaningful Patient Involvement in Medical Education. Academic medicine-philadelphia-. 2017;92(5):575-81.
5. Boelen C, Heck JE, World Health O. Defining and measuring the social accountability of medical schools. Geneva: World Health Organization; 1995.
6. Towle A, Farrell C, Gaines ME, Godolphin W, John G, Kline C, et al. The patient's voice in health and social care professional education: the Vancouver Statement. International Journal of Health Governance. 2016;21(1):18-25.
7. Department of H. Involving patients and the public in healthcare: A discussion document. Department of Health London; 2001.
8. Spencer J, Blackmore D, Heard S, McCrorie P, McHaffie D, Scherpbier A, et al. Patient-oriented learning: a review of the role of the patient in the education of medical students. Medical education. 2000;34(10):851-7.
9. BMA. Medical Education Subcommittee. Role of the patient in medical education. British Medical Association. 2008.
10. Tew J, Gell C, Foster S. Learning from experience: Involving service users and carers in mental health education and training: University of Nottingham, School of Sociology & Social Policy; 2004.
11. Howe A, Anderson J. Involving patients in medical education. Bmj. 2003;327(7410):326-8.
12. Hasman A, Coulter A, Askham J. Education for partnership. Developments in medical education. 2006.
13. **Health Do**. The expert patient: a new approach to chronic disease management for the 21st century. Great Britain: Department of Health; 2001.
14. Organizations IAoP. What is Patient-Centered Healthcare? A Review of Definitions and Principles. London, United Kingdom: IAPO; 2007.
15. Rimmer A. Patients to be asked what they need from doctors. Bmj. 2019;366:l4690.
16. Department of H. The NHS improvement plan: Putting people at the heart of public services: DoH; 2004.
17. Jha V, Setna Z, Al-Hity A, Quinton ND, Roberts TE. Patient involvement in teaching and assessing intimate examination skills: a systematic review. Medical education. 2010;44(4):347-57.
18. Jha V, Quinton ND, Bekker HL, Roberts TE. Strategies and interventions for the involvement of real patients in medical education: a systematic review. Medical education. 2009;43(1):10-20.
19. Gordon M, Gupta S, Thornton D, Reid M, Mallen E, Melling A. Patient/service user involvement in medical education: A best evidence medical education (BEME) systematic review: BEME Guide No. 58. Medical Teacher. 2020;42(1):4-16.
20. Morgan A, Jones D. Perceptions of service user and carer involvement in healthcare education and impact on students' knowledge and practice: a literature review. Medical Teacher. 2009;31(2):82-95.
21. Hong QN, Fàbregues S, Bartlett G, Boardman F, Cargo M, Dagenais P, et al. The Mixed Methods Appraisal Tool (MMAT) version 2018 for information professionals and researchers. Education for Information. 2018;34(4):285-91.
22. Frank JR, Snell L, Sherbino J, Boucher A. CanMEDS 2015. Physician Competency Framework Series I. 2015.
23. Cooper H, Spencer-Dawe E. Involving service users in interprofessional education narrowing the gap between theory and practice. Journal of Interprofessional Care. 2006;20(6):603-17.
24. Doucet S, Lauckner H, Wells S. Patients' messages as educators in an interprofessional health education program. Journal of Research in Interprofessional Practice and Education. 2013;3(1).
25. Council AM. Assessment and Accreditation of Medical Schools. Standards and procedures. 2002.
26. O'Keefe M, Jones A. Promoting lay participation in medical school curriculum development: lay and faculty perceptions. Medical education. 2007;41(2):130-7.
27. Collins L, Arenson C, Jerpbak C, Kane P, Dressel R, Antony R. Transforming chronic illness care education: A longitudinal interprofessional mentorship curriculum. Journal of Interprofessional Care. 2011;25(3):228-30.

For peer review only - http://bmjopen.bmj.com/site/about/guidelines.xhtml

28. Owen C, Reay RE. Consumers as tutors–legitimate teachers? BMC Medical Education. 2004;4(1):14.
29. Towle A, Brown H, Hofley C, Kerston RP, Lyons H, Walsh C. The expert patient as teacher: an interprofessional Health Mentors programme. The Clinical Teacher. 2014;11(4):301-6.
30. Ivory K, Bandler L, Hawke C, Armstrong B. A clinical approach to population medicine. The clinical teacher. 2013;10(2):94-8.
31. McKinlay E, McBain L, Gray B. Teaching and learning about chronic conditions management for undergraduate medical students: utilizing the patient-as-teacher approach. Chronic illness. 2009;5(3):209-18.
32. Stagg P, Rosenthal DR. Why community members want to participate in the selection of students into medical school. Rural & Remote Health. 2012;12(3).
33. Hudson GL, Maar M. Faculty analysis of distributed medical education in Northern Canadian Aboriginal communities. Rural & Remote Health. 2014;14(4).
34. Nestel D, Gray K, Simmons M, Pritchard SA, Islam R, Eng WQ, et al. community perceptions of a rural medical school: a pilot qualitative study. Advances in medical education and practice. 2014;5:407.
35. Fritz C, Naylor K, Watkins Y, Britt T, Hinton L, Curry G, et al. Are we missing the mark? The implementation of community based participatory education in cancer disparities curriculum development. Journal of racial and ethnic health disparities. 2015;2(2):237-43.
36. Noonan EJ, Sawning S, Combs R, Weingartner LA, Martin LJ, Jones VF, et al. Engaging the transgender community to improve medical education and prioritize healthcare initiatives. Teaching and learning in medicine. 2018;30(2):119-32.
37. Kamaka ML. Designing a cultural competency curriculum: asking the stakeholders. Hawaii medical journal. 2010;69(6 Suppl 3):31.
38. Cheng PTM, Towle A. How patient educators help students to learn: an exploratory study. Medical teacher. 2017;39(3):308-14.
39. Muir D, Laxton JC. Experts by experience; the views of service user educators providing feedback on medical students' work based assessments. Nurse education today. 2012;32(2):146-50.
40. Lauckner H, Doucet S, Wells S. Patients as educators: the challenges and benefits of sharing experiences with students. Medical education. 2012;46(10):992-1000.
41. Oswald A, Czupryn J, Wiseman J, Snell L. Patient-centred education: what do students think? Medical education. 2014;48(2):170-80.
42. Oswald AE, Bell MJ, Wiseman J, Snell L. The impact of trained patient educators on musculoskeletal clinical skills attainment in pre-clerkship medical students. BMC medical education. 2011;11(1):65.
43. Hatem DS, Gallagher D, Frankel R. Challenges and opportunities for patients with HIV who educate health professionals. Teaching and learning in medicine. 2003;15(2):98-105.
44. Jarvis-Selinger S, Ho K, Novak Lauscher H, Liman Y, Stacy E, Woollard R, et al. Social accountability in action: University-community collaboration in the development of an interprofessional Aboriginal health elective. Journal of Interprofessional Care. 2008;22(sup1):61-72.
45. Baral K, Allison J, Upadhyay S, Bhandary S, Shrestha S, Renouf T. Rural Community as Context and Teacher for Health Professions Education. Cureus. 2016;8(11).
46. Baral KP, Upadhyay SK, Bhandhary S, Gongal RN, Karki A. Development of community based learning and education system within undergraduate medical curriculum of Patan Academy of Health Sciences. Journal of Nepal Health Research Council. 2016.
47. Haq I, Fuller J, Dacre J. The use of patient partners with back pain to teach undergraduate medical students. Rheumatology. 2005;45(4):430-4.
48. Duggan A, Bradshaw YS, Carroll SE, Rattigan SH, Altman W. What can I learn from this interaction? A qualitative analysis of medical student self-reflection and learning in a standardized patient exercise about disability. Journal of Health Communication. 2009;14(8):797-811.
49. Towle A, Godolphin W. Patients as educators: interprofessional learning for patient-centred care. Medical teacher. 2013;35(3):219-25.
50. Dogra N, Anderson J, Edwards R, Cavendish S. Service user perspectives about their roles in undergraduate medical training about mental health. Medical Teacher. 2008;30(6):e152-e6.
51. Lazarus PA. Patients' experiences and perceptions of medical student candidates sitting a finals examination. Medical Teacher. 2007;29(5):484-9.
52. Jackson A, Blaxter L, Lewando-Hundt G. Participating in medical education: views of patients and carers living in deprived communities. Medical Education. 2003;37(6):532-8.
53. Bokken L, Rethans J-J, Jöbsis Q, Duvivier R, Scherpbier A, van der Vleuten C. Instructiveness of real patients and simulated patients in undergraduate medical education: a randomized experiment. Academic Medicine. 2010;85(1):148-54.
54. Raj N, Badcock LJ, Brown GA, Deighton CM, O'Reilly SC. Undergraduate musculoskeletal examination teaching by trained patient educators—a comparison with doctor-led teaching. Rheumatology. 2006;45(11):1404-8.
55. Gaver A, Borkan JM, Weingarten MA. Illness in context and families as teachers: a year-long project for medical students. Academic Medicine. 2005;80(5):448-51.
56. Bideau M, Guerne P-A, Bianchi M-P, Huber P. Benefits of a programme taking advantage of patient-instructors to teach and assess musculoskeletal skills in medical students. Annals of the rheumatic diseases. 2006;65(12):1626-30.
57. Jain S, Foster E, Biery N, Boyle V. Patients with disabilities as teachers. Fam Med. 2013;45(1):37-9.
58. Oswald AE, Wiseman J, Bell MJ, Snell L. Musculoskeletal examination teaching by patients versus physicians: how are they different? Neither better nor worse, but complementary. Medical teacher. 2011;33(5):e227-e35.
59. de Boer A, Melchers D, Vink S, Dekker F, Beaart L, de Jong Z. Real patient learning integrated in a preclinical block musculoskeletal disorders. Does it make a difference? Clinical rheumatology. 2011;30(8):1029-37.
60. Jaworsky D, Gardner S, Thorne JG, Sharma M, McNaughton N, Paddock S, et al. The role of people living with HIV as patient instructors–reducing stigma and improving interest around HIV care among medical students. AIDS care. 2017;29(4):524-31.
61. Barr J, Ogden K, Rooney K. Committing to patient-centred medical education. The clinical teacher. 2014;11(7):503-6.
62. Humphrey-Murto S, Smith CD, Touchie C, Wood TC. Teaching the musculoskeletal examination: are patient educators as effective as rheumatology faculty? Teaching and learning in medicine. 2004;16(2):175-80.
63. Saketkoo L, Anderson D, Rice J, Rogan A, Lazarus CJ. DEVELOPMENTS: Effects of a Disability Awareness and Skills Training Workshop on Senior Medical Students as Assessed with Self Ratings and Performance on a Standardized Patient Case. Teaching and Learning in Medicine. 2004;16(4):345-54.

For peer review only - http://bmjopen.bmj.com/site/about/guidelines.xhtml

64. Langlois S, Lymer E. Learning professional ethics: Student experiences in a health mentor program. Education for Health. 2016;29(1):10.
65. Coret A, Boyd K, Hobbs K, Zazulak J, McConnell M. Patient Narratives as a Teaching Tool: A Pilot Study of First-Year Medical Students and Patient Educators Affected by Intellectual/Developmental Disabilities. Teaching and learning in medicine. 2018;30(3):317-27.
66. Jha V, Quinton ND, Bekker HL, Roberts TE. What educators and students really think about using patients as teachers in medical education: a qualitative study. Medical education. 2009;43(5):449-56.
67. Henriksen A-H, Ringsted C. Medical students' learning from patient-led teaching: experiential versus biomedical knowledge. Advances in Health Sciences Education. 2014;19(1):7-17.
68. Alahlafi A, Burge S. What should undergraduate medical students know about psoriasis? Involving patients in curriculum development: modified Delphi technique. Bmj. 2005;330(7492):633-6.
69. Tierney WM. Use of Stakeholder Focus Groups to Define the Mission and Scope of a new Department of Population Health. Journal of general internal medicine. 2018;33(7):1069-76.
70. Wagner AC, Hart TA, McShane KE, Margolese S, Girard TA. Health care provider attitudes and beliefs about people living with HIV: Initial validation of the health care provider HIV/AIDS Stigma Scale (HPASS). AIDS and Behavior. 2014;18(12):2397-408.
71. Rees CE, Knight LV, Wilkinson CE. "User involvement is a sine qua non, almost, in medical education": learning with rather than just about health and social care service users. Advances in Health Sciences Education. 2007;12(3):359-90.
72. Lucas B, Pearson D. Patient perceptions of their role in undergraduate medical education within a primary care teaching practice. Education for Primary Care. 2012;23(4):277-85.
73. Gutteridge R, Dobbins K. Service user and carer involvement in learning and teaching: A faculty of health staff perspective. Nurse education today. 2010;30(6):509-14.

Box 3: Practical points for patient organizations wishing to engage in medical education

**Practical points for patient organizations**
♦ Highlight potential benefits of involvement in medical education to members and respond to common motivations<br>♦ Facilitate peer support networks among patients involved in medical education<br>♦ Investigate whether teaching facilities in your area have existing frameworks for patients and public involvement, have made explicit commitments to patient and public involvement and/or have appointed officers specifically assigned to patient and public involvement that could provide you with a starting point for collaboration<br>♦ Call for the creation of a patient committee or advisory group within a medical faculty to enable and safeguard structural input into the educational process<br>♦ Determine which step of the educational process is best suited for your goals. You might choose to focus your efforts on the mission and vision of the school, learning objectives, educational strategies, teaching, assessment of learners or the evaluation of the course.<br>♦ Not every meaningful intervention has to be at an overall faculty level, they may also be on specific areas such as one learning objective or a disease-specific course.<br>♦ Showcase successful collaborations with universities and best practices to the wider patient and academic communities

Box 4: Spectrum of patient involvement in medical education by Towle et al. (2010)

**Spectrum of patient involvement in medical education as defined by Towle et al. (2010)**
1. Patients as focus of a paper-based or electronic cases<br>2. Standardized or volunteer patients in clinical settings<br>3. Patients sharing their experiences with students in a faculty directed curriculum<br>4. Patient-teachers involved in teaching or evaluating students<br>5. Patient-teachers as equal partners in student education, evaluation and curriculum development beyond specific courses, to the curriculum as a whole<br>6. Patients involved at the institutional level in addition to sustained involvement as patient-teachers in education, evaluation and curriculum development.

SUPPLEMENTARY FILE 1: Full search strategy

SUPPLEMENTARY FILE 2: Quality assessment

SUPPLEMENTARY FILE 3: Study characteristics

FIGURE 1: Results of the systematic literature search

For peer review only - http://bmjopen.bmj.com/site/about/guidelines.xhtml

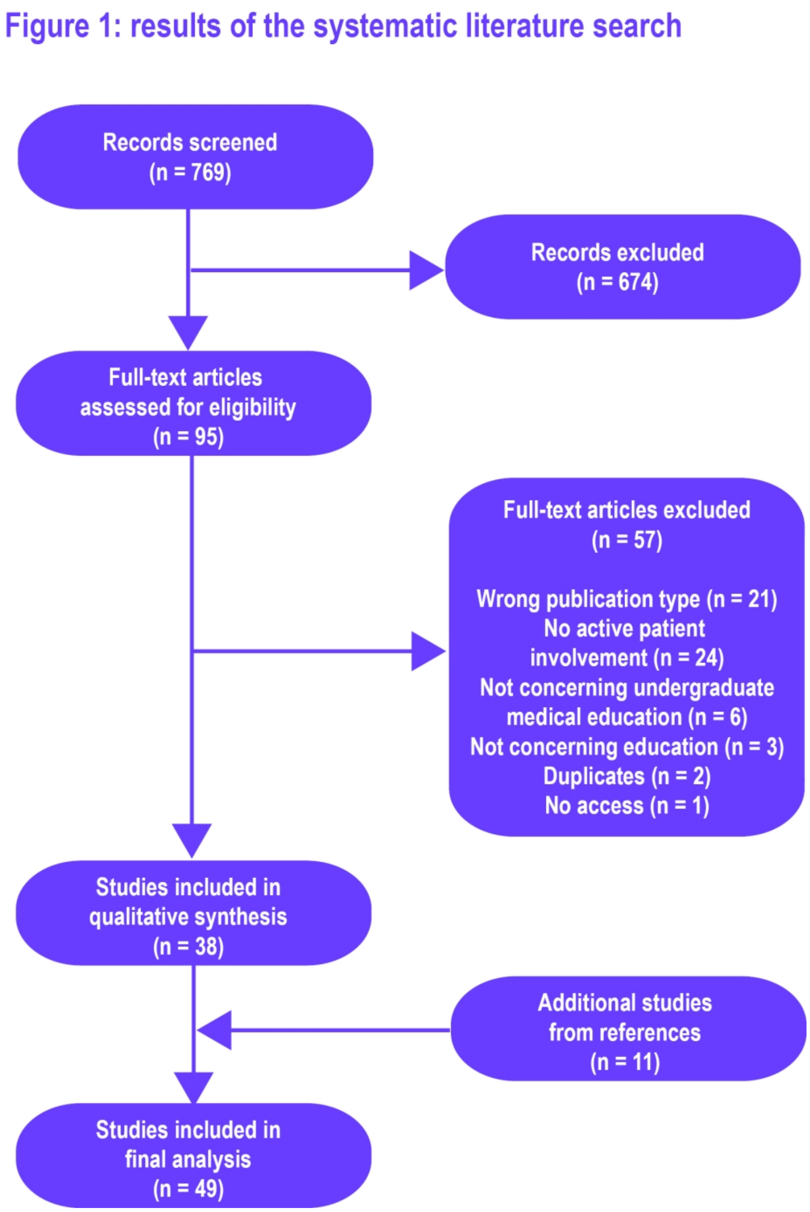

Figure 1: Study flowchart

139x207mm (300 x 300 DPI)

Full search strategy

The authors performed a search through PubMed on July 12th 2018. The following search terms were used:

((“Patient Participation”[Mesh] OR "Community Participation"[Mesh] OR “Stakeholder Participation”[Mesh] OR “patient instructors”[tiab] OR “patient educators”[tiab] OR “patient teachers”[tiab] OR “patient-as-teacher”[tiab] OR patient participation [tiab] OR patient involvement [tiab] OR community participation [tiab] OR community involvement [tiab] OR patient engag [tiab] OR community engag* [tiab] OR patient cooperat* [tiab] OR community cooper* [tiab] OR patient collaborat* [tiab] OR community collaborat* [tiab] OR patient represent* [tiab] OR community represent* [tiab])*

AND

(“Education, Medical, Undergraduate”[Mesh] OR "Education, Medical"[Mesh] OR “Curriculum”[Mesh] OR “Students, Medical”[Mesh] OR medical education [tiab] OR medical student [tiab]))*

A limit was set to only show articles written in English and published between 2003 and 2018 to be included. Exclusion criteria were studies that did not address education to undergraduate medical students, where patients were only passively involved in education (for example, through presence at wards). Articles were first assessed based on title and abstract, and in a second round on full text. The references of all included articles were assessed for additional studies that matched the original inclusion criteria. All reviews that complied with the inclusion criteria were additionally assessed for relevant references, however, only original research articles were included.

For peer review only

Journal characteristics				SCREENING QUESTIONS		1. Q	
Authors	Year of publication	Journal	Study design	S1. Are there clear research questions?	S2. Do the collected data allow to address the research questions?	1.1. Is the qualitative approach appropriate to answer the research	1.2. Are the qualitative data collection methods adequate to address the research
Rees, C.E., Knight, L.V., Wilkinson, C.E.	2006	Advances in Health Sciences Education	Qualitative description	Yes	Yes	Yes	Yes
Ivory, K. et al.	2013	The Clinical Teacher	Case study	No	Can't tell	Can't tell	Can't tell
Fritz, C. et al.	2015	Journal of Racial and Ethnic Health Disparities	Mixed methods - Exploratory design	Yes	Yes	Yes	Yes
Bideau, M et al.	2006	Annals of the rheumatic diseases	Before and after study	Yes	Yes		
Hatem, D.S., Gallagher, D., Frankel, R.	2003	Teaching and Learning in Medicine	Qualitative - Phenomenological design	Yes	Yes	Yes	Yes
Barr, J. et al.	2014	The Clinical Teacher	Case study	No	Can't tell	Can't tell	Can't tell
Nestel, D. et al.	2014	Advances in Medical Education and Practice	Qualitative description	Yes	Yes	Yes	Yes
Owen, C., Reay, R.	2004	BMC Medical Education	Mixed methods - Convergent design	Yes	Yes	Yes	Yes
Kamaka, M.L.	2010	Hawai'i Medical Journal	Qualitative description	Yes	Yes	Yes	Yes
Baral, K.P., et al.	2016	Journal of Nepal Health Research Council	Case study	No	Can't tell	Can't tell	Can't tell
Saketkoo, L. et al.	2004	Teaching and Learning in Medicine	Case-Control study	Yes	Yes		

Noonan E.J. et al.	2018	Teaching and learning in medicine.	Qualitative - Grounded theory design	Yes	Yes	Yes	Yes
Muir, D., Laxton, J.C.	2012	Nurse Education Today	Case study	Yes	Yes	Yes	Yes
Hudson, G.L., Maar, M.	2014	Rural and Remote Health	Case study	No	Can't tell	Can't tell	Can't tell
Cheng, P., Towle, A.	2017	Medical Teacher	Qualitative - Exploratory design	Yes	Yes	Yes	Yes
Gaver, A. et al.	2005	Academic Medicine	Case study	No	Can't tell	Can't tell	Can't tell
Bokken, L. et al.	2010	Academic Medicine	Mixed Methods - Explanatory design	Yes	Yes	Yes	Yes
Cooper Director, H., Spencer-Daw, E.	2006	Journal of Interprofessional Care	Case study	No	Can't tell	Can't tell	Can't tell
Langlois, S., Lymer, E.	2016	Education for Health	Qualitative - Phenomenological design	Yes	Yes	Yes	Yes
Henriksen, A., Ringsted, C.	2014	Advances in Health Sciences Education: Theory and Practice	Qualitative - Grounded theory design	Yes	Yes	Yes	Yes
Oswald, A.E. et al.	2011 b	Medical Teacher	Case study	Yes	Yes	Yes	Yes
Jackson, A., Blaxter, L., Lewando-Hundt, G.	2003	Medical Education	Qualitative - Phenomenological design	Yes	Yes	Yes	Yes
Coret, A. et al.	2018	Teaching and Learning in Medicine	Mixed methods - Convergent design	Yes	Yes	Yes	Yes
Lucas, B, Pearson, D.	2012	Education for Primary Care	Case study	Yes	Yes	Yes	Yes

Oswald, A.E. et al.	2014	Medical Education	Qualitative - Grounded theory design	Yes	Yes	Yes	Yes
Towle, A., Godol	2013	Medical Teacher	Case study	Yes	Yes	Yes	Yes
Lauckner, H., Do	2012	Medical Education	Qualitative description	Yes	Yes	Yes	Yes
Jain, S. et al.	2013	Family Medicine	Case study	No	Can't tell	Can't tell	Can't tell
Lazarus, P.A.	2007	Medical Teacher	Qualitative description	Yes	Yes	Yes	Yes
Doucet, S., Lauc	2013	Journal of Researc	Qualitative description	Yes	Yes	Yes	Yes
O'Keefe, M., Jon	2007	Medical Education	Qualitative description	Yes	Yes	Yes	Yes
de Boer, A. et al.	2011	Clinical Rheumatol	Mixed model - convergent d	Yes	Yes	Yes	Yes
Baral, K. et al.	2016	Cureus	Case study	No	Can't tell	Can't tell	Can't tell
Gutteridge, R., D	2009	Nurse Education To	Qualitative - Grounded theory design	Yes	Yes	Yes	Yes
Dogra, N. et al.	2008	Medical Teacher	Qualitative description	Yes	Yes	Yes	Yes
Jarvis-Selinger, S	2008	Journal of Interprof	Case study	No	Can't tell	Can't tell	Can't tell
McKinlay, E, McE	2009	Chronic Illness	Qualitative description	Yes	Yes	Yes	No

Humphrey-Murto	2004	Teaching and Learn	Randomized controlled clinic	Yes	Yes		
Towle, A. et al.	2014	The Clinical Teache	Case study	No	Can't tell	Can't tell	Can't tell
Oswald, A.E. e.a	2011 a	BMC Medical Educ	Randomized controlled clinic	Yes	Yes		
Jaworsky, D. et a	2017	AIDS Care	Before and after study	Yes	Yes		
Haq, I. Fuller, J.,	2006	Rheumatology	Randomized controlled clinic	Yes	Yes		
Collins, L. et al.	2011	Journal of Interprof	Qualitative description	Yes	Yes	Yes	Yes
Raj, N. et al.	2006	Rheumatology	Randomized controlled clinic	Yes	Yes		
Tierney, W.M.	2018	Journal of General	Case study	No	Can't tell	Can't tell	Can't tell
Duggan, A. et al.	2009	Journal of Health C	Qualitative - Grounded theory design	Yes	Yes	Yes	Yes
Jha, V. et al.	2009	Medical Education	Qualitative description	Yes	Yes	Yes	Yes
Alahlafi, A., Burg	2005	British Medical Jou	Case study	No	Can't tell	Can't tell	Can't tell
Stagg, P., Rosen	2012	Rural and Remote	Qualitative description	Yes	Yes	Yes	Yes

Number of items: yes	37	37	30	29
Number of items: can't tell	0	12	12	12
Number of items: no	12	0	0	1
% Yes	0.755102041	0.755102041	0.714285714	0.69047619

For peer review only

QUALITATIVE STUDIES			2. RANDOMIZED CONTROLLED TRIALS				
1.3. Are the findings adequately derived from the data?	1.4. Is the interpretation of results sufficiently substantiated by data?	1.5. Is there coherence between qualitative data sources, collection, analysis	2.1. Is randomization appropriately performed?	2.2. Are the groups comparable at baseline?	2.3. Are there complete outcome data?	2.4. Are outcome assessors blinded to the intervention provided?	2.5 Did the participants adhere to the assigned intervention?
Yes	Yes	Yes					
Can't tell	Can't tell	Can't tell					
Yes	Nyes	Yes					
Yes	Yes	Yes					
Can't tell	Can't tell	Can't tell					
Yes	Yes	Yes					
Yes	Yes	Yes					
Yes	Yes	Yes					
Can't tell	Can't tell	Can't tell					

For peer review only

Yes	Yes	Yes					
Yes	Yes	Yes					
Can't tell	Can't tell	Can't tell					
Yes	Yes	Yes					
Can't tell	Can't tell	Can't tell					
Yes	Yes	Yes	Can't tell	Can't tell	No	No	Yes
Can't tell	Can't tell	Can't tell					
Yes	Yes	Yes					
Yes	Yes	Yes					
Yes	Yes	Yes					
Yes	Yes	Yes					
Yes	Yes	Yes	Can't tell	Can't tell	Yes	Yes	Yes
Yes	Yes	Yes					

review only

Yes	Yes	Yes
No	No	No
Yes	Yes	Yes
Can't tell	Can't tell	Can't tell
Yes	No	No
Yes	No	Yes
Yes	No	No
No	No	No
Can't tell	Can't tell	Can't tell
Yes	Yes	Yes
Yes	Yes	Yes
Can't tell	Can't tell	Can't tell
Yes	No	No

For peer review only

			Can't tell	Can't tell	Yes	No	Yes
Can't tell	Can't tell	Can't tell					
			No	Can't tell	Yes	No	Yes
			No	Can't tell	Yes	No	Yes
Can't tell	No	No					
			Yes	Yes	Yes	Yes	Yes
Can't tell	Can't tell	Can't tell					
Yes	Yes	Yes					
Yes	Yes	Yes					
Can't tell	Can't tell	Can't tell					
Yes	Yes	Yes					

For peer review only

27	22	24	1	1	5	2	6
13	12	12	3	5	0	0	0
2	7	6	2	0	1	4	0
0.642857143	0.536585366	0.571428571	0.166666667	0.166666667	0.833333333	0.333333333	1

For peer review only

3. NON-RANDOMIZED STUDIES					4. QUANTITATIVE DESCRIPTIV		
3.1. Are the participants representative of the target population?	3.2. Are measurements appropriate regarding both the outcome and intervention (or	3.3. Are there complete outcome data?	3.4. Are the confounders accounted for in the design and analysis?	3.5. During the study period, is the intervention administered (or exposure occurred) as	4.1. Is the sampling strategy relevant to address the research question?	4.2. Is the sample representative of the target population?	4.3. Are the measurements appropriate?
					Yes	No	Yes
Yes	Yes	Yes	No	Yes			
					Yes	Yes	Yes
					Yes	No	Yes

For peer review only

Yes	Yes	Yes	No	Yes

For peer review only

Yes	Yes	Yes	No	Yes

For peer review only

3	3	3	0	3	3	1	3
0	0	0	0	0	0	0	0
0	0	0	3	0	0	2	0
1	1	1	0	1	1	0.333333333	1

For peer review only

VE STUDIES		5. MIXED METHODS STUDIES					
4.4. Is the risk of nonresponse bias low?	4.5. Is the statistical analysis appropriate to answer the research question?	5.1. Is there an adequate rationale for using a mixed methods design to address the research	5.2. Are the different components of the study effectively integrated to answer the	5.3. Are the outputs of the integration of qualitative and quantitative components	5.4. Are divergences and inconsistencies between quantitative and qualitative results	5.5. Do the different components of the study adhere to the quality criteria of each tradition of	COMMENTS
No	No	Yes	Yes	Yes	Yes	No	The effects of bias are not discussed. No statistical analysis of response data.
No	Yes	Yes	Yes	No	No	No	Negative trends in student responses are not taken into account in the qualitative analysis. Missing
No	Yes						

For peer review only

Yes	Yes	Yes	Yes	No	
Yes	Yes	No	Yes	No	No statistical analysis of difference between groups. No information comparability

For peer review only

Yes	Yes	no	Yes	no

For peer review only

For peer review only

0	2	5	5	2	4	0
0	0	0	0	0	0	0
3	1	0	0	3	1	5
0	0.666666667	1	1	0.4	0.8	0

For peer review only

Number of "Yes"	Number of "Can't tell"	Number of "No"	Papers with no concerns (0) or with some concern (>0)
7	0	0	0
0	0	5	5
12	4	0	4
6	1	0	1
7	0	0	0
0	0	5	5
7	0	0	0
13	4	0	4
7	0	0	0
0	0	5	5
5	2	0	2

For peer review only

7	0	0	0
7	0	0	0
0	0	5	5
7	0	0	0
0	0	5	5
12	3	2	5
0	0	5	5
7	0	0	0
7	0	0	0
7	0	0	0
7	0	0	0
13	2	2	4
7	0	0	0

For peer review only

7	0	0	0
4	3	0	3
7	0	0	0
0	0	5	5
5	2	0	2
6	1	0	1
5	2	0	2
11	6	0	6
0	0	5	5
7	0	0	0
7	0	0	0
0	0	5	5
4	3	0	3

For peer review only

4	1	2	3
0	0	5	5
4	2	1	3
6	1	0	1
4	2	1	3
4	2	1	3
7	0	0	0
0	0	5	5
7	0	0	0
7	0	0	0
0	0	5	5
7	0	0	0
		Papers with no concern	20

For peer review only

Papers with some concern	29
Percentage no concern	0.408163265

For peer review only

Study	D1	D2	D3	D4	D5	D6
Rees 2006	Low	Low	Low	Low	Low	Low
Ivory 2013	High	Some concerns	Some concerns	Some concerns	Some concerns	Some concerns
Fritz 2015	Low	Low	Low	Low	Low	Some concerns
Bideau 2006	Low	Low	Low	Low	Low	High

For peer review only

For peer review only

D7
Low
Some concerns
High
Low

For peer review only

Authors	Year of publication	Journal	Title	Country	Resarch question	Study design	Data collection	Setting	Outcome measure	# Patients in intervention	Patient characteristics	Role of patient organizations	Type of patient engagement	# Students in intervention
**Rees, C.E., Knight, L.V., Wilkinson, C.E.**	2006	Advances in Health Sciences Education	''User Involvement Is a Sine Qua Non, Almost, in Medical Education'': Learning with Rather than Just About Health and Social Care Service Users	UK	What are the views and experiences of stakeholders concerning user involvement in medical education?	Qualitative description	Focus groups	Medical school - general	Service user views and experiences on service user involvement in medical education	19	Health or social care service users	Medical educators explicitly preferred not to work with politicized groups of patients, but did include groups to hear multiple voices. Service users wanted to participate in groups in order to receive support.	Share views on service user involvement	
**Ivory, K. et al.**	2013	The Clinical Teacher	A clinical approach to population medicine	Australia	Not specified	Case study	Programme evaluation	Medical school - general	Description of the development of an integrated, community engaged population medicine curriculum		Patients with chronic health conditions	Partnership with consumer organizations to recruit patients	Contact with individual students during a six month period	
**Fritz, C. et al.**	2015	Journal of Racial and Ethnic Health Disparities	Are We Missing the Mark? The Implementation of Community Based Participatory Education in Cancer Disparities Curriculum Development	USA	What is the interest of community members in participating in curriculum design, how should they be involved in designing a cancer disparities curriculum, and what do community members believe the curriculum should address to positively impact their community?	Mixed methods - Exploratory design	Deliberative session and survey	Community forum	Stakeholder perspectives on creation of a cancer disparities curriculum for medical students and public health students	86	Members of Chicago community (focus on cancer)	Study concept directly derived from cancer support group	Curriculum development	
**Bideau, M et al.**	2006	Annals of the rheumatic diseases	Benefits of a programme taking advantage of patient-instructors to teach and assess musculoskeletal skills in medical students.	Switzerland	How effective is a rheumatid arthritis patient-instructor-based formation-assessment programme for its ability to improve and assess musculoskeletal knowledge and skills in third-year medical students?	Before and after study	Surveys and performance records	Patient-instructor programme to improve and assess musculoskeletal skills	Observed and reported student performance	11	All women, aged 29–66 years, who have had rheumatoid arthritis for at least 5 years.	Involved in patient recruitment	Active teacher, student assessor, feedback/coach	61
**Hatem, D.S., Gallagher, D., Frankel, R.**	2003	Teaching and Learning in Medicine	Challenges and Opportunities for Patients With HIV Who Educate Health Professionals	USA	What are the personal and programmatic effects of using HIV-infected persons as teachers in courses about care of HIV-infected people?	Qualitative - Phenomenological design	Interviews using semi-structured questionnaires	Course on HIV infection in which persons living with HIV cofacilitate small-group teaching and participate in panels	Patients responses on their motivation ad experiences	8	People living with HIV/AIDS, age 26-50		Curriculum development, development of learning material, active teacher	
**Barr, J. et al.**	2014	The Clinical Teacher	Committing to patient-centred education	Australia	Not specified	Case study	Programme evaluation	Programme where patient partners and students meet in weekly small group learning sessions facilitated by a clinician	Description of the program, implications, impact on learning, challenges		Patients from community practices (specialist and general)		Share experiences, give feedback on students' interviewing skills	
**Nestel, D. et al.**	2014	Advances in Medical Education and Practice	Community percpetions of a rural medical school: a pilot qualitative study	Australia	In what ways can [the medical school] engage with its local community? What are the community's expectations of [the medical school's] students hile completing their medical degree and upon graduaton?	Qualitative description	Interviews	Medical school - general	Expectations of lay community members	12	Any member of lay public, recruited through local newspaper advertisement		Interviewed to share perspective on community engagement	
**Owen, C., Reay, R.**	2004	BMC Medical Education	Consumers as tutors - legitimate teachers?	Australia	What is the feasibility of training mental health consumers as tutors for 4th year medical students in psychiatry?	Mixed methods - Convergent design	Programme evaluation	Programme where patients are trained as tutors and deliver inter a curriculum of interviewing skills	Patient, student and tutor feedback on their experience and attitudes	20	Consumers of mental health services	Partner in steering group and recruitment	Active patient teacher, curriculum development	104
**Kamaka, M.L.**	2010	Hawai'i Medical Journal	Designing a Cultural Competency Curriculum: Asking the Stakeholders	USA	What components should be included in a curriculum targeting Native Hawaiians?	Qualitative description	Focus groups	Medical school - general	Themes resulting from community focus grous	34 stakeholders (including students and physiicians)	Native Hawai'ians		Curriculum development	
**Baral, K.P., et al.**	2016	Journal of Nepal Health Research Council	Development of Community Based Learning and Education system within Undergraduate Medical Curriculum of Patan Academy of Health Sciences	Nepal	Not specified	Case study	Description of a curriculum development process	Medical school - general	A description and framework of curriculum development		Community members hosting students during field stay, local community leaders and local health workers involved in assessing students		Feedback/coach, student selection, teacher, assessor	

For peer review only

**Saketkoo, L. et al.**	2004	Teaching and Learning in Medicine	Effects of a disability awareness and skills training workshop on senior medical students as assessed with self ratings and performance on a standardized patient case	USA	What is the efficacy of a 3 hour workshop for medical students on disability skills and awareness?	Case-Control study	Surveys	A disability awareness and skills training workshop	Student self-efficacy ratings, knowledge of disability resources and medical complications, attitudes, confidence in ability to effectively advocate for patients with disabilities	3	1 team member with visual impairment, 1 attorney with mental illness, 1 parent of a nonverbal child	Partner in organizing team	Active teacher	147
**Noonan E.J. et al.**	2018	Teaching and learning in medicine.	Engaging the Transgender Community to Improve Medical Education and Prioritize Healthcare Initiatives	USA	What are common perceptions of transgender healthcare, priorities for improvement interventions, and themes to inform the curriculum?	Qualitative - Grounded theory design	Forum discussions with follow up questionnaire	Community forum	LGBT community views on transgender disparities in medical education	10-15	Transgender	Recruitment via local LGBT organizations	Curriculum development	
**Muir, D., Laxton, J.C.**	2012	Nurse Education Today	Experts by experience; the views of service user educators providing feedback on medical students' work based assessments	UK	What are the experiences of service users providing feedback to students on formative, workbased assessments?	Case study	Focus group with written feedback	Development and application of an assessment tool	Reflections by patient assessors following the analysis of student assessments using an interprofessional assessment tool	6	Lay people with experiences of ill health, caring and accessing health services	Lay group "The Patient Voice Group" of 6 members participated in the project	curriculum development, development of learning material, active teacher, student assessor, feedback/coach	253
**Hudson, G.L., Maar, M.**	2014	Rural and Remote Health	Faculty analysis of distributed medical education in Northern Canadian Aboriginal communities	Canada	Not specified	Case study	Programme evaluation	Mandatory Aboriginal community placement for all first-year medical students	Lessons learned and suggestions for curriculum improvement	7 communities	Aboriginal communities		Curriculum development, student assessor, feedback/coach, student selection	15
**Cheng, P., Towle, A.**	2017	Medical Teacher	How patient educators help students to learn: An exploratory study	Canada	What are the teaching practices and experiences that prepared patient educators for their roles in a longitudinal interprofessional Health Mentors program?	Qualitative - Exploratory design	Semi-structured interviews	Longitudinal interprofessional health mentors program	Experiences of and teaching methods used by interprofessional health mentors	11	Various conditions, 30-60 years		Active teacher	
**Gaver, A. et al.**	2005	Academic Medicine	Illness in context and families as teachers: A year-long project for medical students	Israel	Not specified	Case study	Student essays	Programme where second year students meet a volunteer family five times	Programme evaluation and student learning based on analysis of essays	100	Volunteer family, one of whose members suffers from a chronic disease or other impairment.	Involved in recruitment of families	Active teacher	40
**Bokken, L. et al.**	2010	Academic Medicine	Instructiveness of real patients and simulated patients in undergraduate medical education: a randomized experiment	Netherlands	Which contact (real patient or SP) is perceived as most instructive by students and which variables contribute to this?	Mixed Methods - Explanatory design	Quantitative questionnaires and qualitative focus groups	Volunteer sign up skillslab sessions with patient or SP encounters for first year students	Instructiveness to students (quantitative and qualitative)	9	Patients with asthma (age 54-82)		Active teacher, feedback/coach	163
**Cooper Director, H., Spencer-Daw, E.**	2006	Journal of Interprofessional Care	Involving service users in interprofessional education narrowing the gap between theory and practice	UK	Not specified	Case study	Students' reflective narratives, a focus group with practitioners, and individual semi-structured interviews with service users	Voluntary programme with four interprofessional student group workshops with e-learning and an introductory plenary.	Programme evaluation	35	Health or social care service users	Advise, provide assistance and support	Curriculum development, active teacher	~500
**Langlois, S., Lymer, E.**	2016	Education for Health	Learning professional ethics: Student experiences in a health mentor program	Canada	What are the experiences and learning of health profession students engaged in an ethics module as part of a health mentor program?	Qualitative - Phenomenological design	Student reflective papers and online discussions	Health mentor programme	Student experiences and learning	17	People with chronic health challenges		Active teacher, Feedback/coach	91
**Henriksen, A., Ringsted, C.**	2014	Advances in Health Sciences Education: Theory and Practice	Medical students' learning from patient-led teaching: experiential versus biomedical knowledge	Denmark	How do medical students perceive the experience of learning from ptient instructors in the context of coupled faculty-led and patient-led teaching sessions?	Qualitative - Grounded theory design	Focus group interviews	Medical school - general	Student perceptions	22	Patient Instructors associated with the Danish Association of Rheumatism	Employer of Patient Instructor leader	Curriculum development, development of learning material, active teacher	39

For peer review only

**Oswald, A.E. et al.**	2011 b	Medical Teacher	Musculoskeletal examination teaching by patients versus physicians: how are they different? Neither better nor worse, but complementary	Canada	How are musculoskeletal examination teaching by patients versus physicians different?	Case study	Video recording analysis	Medical school - general	Observed qualitative comparison of features and themes emerging from training	2	Lay people with a chronic MSK condition who undertake formal training in teaching musculo-skeletal physical examination skills		Active teacher	6 small groups
**Jackson, A., Blaxter, L., Lewando-Hundt, G.**	2003	Medical Education	Participating in medical education: views of patients and carers living in deprived communities	UK	What are the views of patients and carers living in deprived areas on their participation in medical education?	Qualitative - Phenomenological design	Semi-structured interviews	Patients' homes	Patient and carer views	54	Patients who live in socially and economically deprived areas, child carers, midlife patients and older adult patients		Active teacher	
**Coret, A. et al.**	2018	Teaching and Learning in Medicine	Patient Narratives as a Teaching Tool: A Pilot Study of First-Year Medical Students and Patient Educators Affected by Intellectual/Developmental Disabilities	Canada	What are trainees' experiences and learning outcomes resulting from exposure to video narratives followed by PE encounters?	Mixed methods - Convergent design	Focus groups, quantitative performance assessments	Video narratives by patients followed by patient-expert encounters	Qualitative and quantitative observed and self-reported data on student performance in clinical encounters	7	People living with intellectual and developmental disabilities (IDD)		Narrator	27
**Lucas, B, Pearson, D.**	2012	Education for Primary Care	Patient perceptions of their role in undergraduate medical education within a primary care teaching practice	UK	What are patients' perception of their role in undergraduate medical education within a UK primary care setting	Case study	Interviews	Primary care	Patients' perceptions	18	Patients who volunteered for involvement with pre-arranged undergraduate clinics		Active teacher	
**Oswald, A.E. et al.**	2014	Medical Education	Patient-centred education: what do students think?	Canada	What themes might be identified through a qualitative analysis of students' reflective writing on patient-centred education? What are common students' perceptions regarding patients as educators?	Qualitative - Grounded theory design	Students' written reflections	Patient Partners in Arthritis programme that teaches 4 hours of the 8 hours of pre-clinical MSK clinical skills small-group sessions	Student reflections on experiences of patient-educators		Volunteer patient educators with arthritis		Active teacher	356
**Towle, A., Godolphin, W.**	2013	Medical Teacher	Patients as educators: Interprofessional learning for patient-centred care	Canada	What issues are involved in creating an educational intervention designed and delivered by patients and document outcomes?	Case study	Focus groups with patient educators and staff, surveys with students	Interprofessional educational workshop focused on chronic disease management	Workshop design, student experiences and learning outcomes	24	Community Educators with epilepsy, arthritis, HIV/AIDS and mental health problems	Community organizations with a health mandate are part of Advisory Group	Curriculum development, development of learning material, active teacher	142
**Lauckner, H., Doucet, S., Wells, S.**	2012	Medical Education	Patients as educators: the challenges and benefits of sharing experiences with students	Canada	What are positive and negative factors that contribute to the experiences of patient-educators?	Qualitative description	Semi-structured focus group discussions and individual interviews	Mentor programme focused on patient - centred care, interprofessional collaboration, and chronic conditions and disabilities.	Conceptual framework of factors impacting patient experience	30	Adult volunteers with chronic conditions or disabilities. Mentors' conditions included diabetes, chronic pain, multiple sclerosis, cancer, stroke and various rare conditions.		Active teacher	
**Jain, S. et al.**	2013	Family Medicine	Patients with Disabilities as Teachers	USA	Not specified	Case study	Surveys with students	One hour session with patient with chronic disease facilitated by a faculty member	Description of programme	2	Persons with disabilities in their late 20's, who use wheelchairs; 1. spinal musclar atrophy, 1 cerebral palsy.		Active teacher	44
**Lazarus, P.A.**	2007	Medical Teacher	Patients' experiences and perceptions of medical student candidates sitting a finals examination	UK	What features of student candidates at finals do patients notice and consider when making judgements on them?	Qualitative description	Focus groups with patients	Finals examination of medical students before entering the clinical phase.	Patient perceptions and opinons of students during final examinatin	10	Patients, who had been involved in final examns, all >30 y.o, all white British ethnicity		Curriculum development and student assessor	
**Doucet, S., Lauckner, H., Wells, S.**	2013	Journal of Research in Interprofessional Practice and Education	Patients' Messages as Educators in an Interprofessional Health Education Program	Canada	What motivates patient educators to participate in a mentor programme and what messages they wanted to instill?	Qualitative description	Semi-structured focus groups	Mentor programme focused on patient - centred care, interprofessional collaboration, and chronic conditions and disabilities.	Thematic experiences of patient-educators	30	Community volunteers with a chronic condition and/or disability	Recruitment via patient networks	Active teacher	

For peer review only

**O'Keefe, M., Jones, A.**	2007	Medical Education	Promoting lay participation in medical school curriculum development: lay and faculty perceptions	Australia and NZ	What are lay and faculty perceptions around lay participation in medical curriculum development and how do they compare?	Qualitative description	Focus groups	Medical school - general	Identification of challenges of lay participation in medical curriculum development	17	Lay public		Curriculum development	
**de Boer, A. et al.**	2011	Clinical Rheumatology	Real patient learning integrated into a preclinical block musculosceletal disorders. Does it make a difference?	Netherlands	What are the educational effectiveness of the practicals, the expectations students have of real patients, and students' satisfaction?	Mixed model - convergent design	Formal written tests, surveys, focus groups	Non-obligatory practicals during a preclinical block on musculoskeletal disorders.	Educational effectiveness, student opinions and expectations, experienced effects	>4	Patients of the departments of rheumatology, orthopedic surgery, and/or patients under treatment of the local physical therapists.	Patients with spondylarthritis are members of a professional organization of arthritis educators which employs patients with RA in different phases of their disease.	Active teacher	377
**Baral, K. et al.**	2016	Cureus	Rural Community as Context and Teacher for Health Professions Education	Nepal	Not specified	Case study	Description of curriculum development	Describing an approach of ruralizing the academic medical education	Description of the program		Community members and leaders from rural areas, women's groups, CBOs, red cross, local health institutions		Curriculum development, selection of students, setting of school mission and vision, teaching	
**Gutteridge, R., Dobbins, K.**	2009	Nurse Education Today	Service user and carer involvement in learning and teaching: A faculty of health staff perspective	UK	What are staff views on current and ideal patient involvement?	Qualitative - Grounded theory design	Semi-structured interviews	Medical school - general	Academic and administrative staff views					
**Dogra, N. et al.**	2008	Medical Teacher	Service user perspectives about their roles in undergraduate medical training about mental health	UK	What are user perspectives on the specific role of service users in the delivery of teaching psychiatry?	Qualitative description	Focus groups	Medical school - general	Service user perspectives	28	16 men 12 women, all white adults, one of them a carer		Curriculum development	
**Jarvis-Selinger, S. et al.**	2008	Journal of Interprofessional Care	Social accountability in action: University-community collaboration in the development of an interprofessional Aboriginal health elective	Canada	Not specified	Case study	Description of curriculum development	Co-development of an Aboriginal Health course	Recommendations for curriculum development, issues and experiences	2 communities	Aboriginal leaders, instructors and Elders		Curriculum development	7
**McKinlay, E, McBain, L., Gray, B.**	2009	Chronic Illness	Teaching and learning about chronic conditions management for undergraduate medical students: utilizing the patient-as-teacher approach	NZ	What is the impact on medical student learning of a revised chronic conditions teaching programme?	Qualitative description	Surveys, reflective assays	Chronic conditions teaching programme based on the chronic care model utilizing	Student feedback on involvement of patients in teaching/home visit (before/after)		Patients with a specific, usually physical, chronic condition (such as diabetes)		Active teacher	57
**Humphrey-Murto, S. et al.**	2004	Teaching and Learning in Medicine	Teaching the Musculoskeletal Examination: Are Patient Educators as effective as Rheumatology Faculty?	Canada	What is the teaching effectiveness of patient educators compared to rheumatology faculty?	Randomized controlled clinical trial	A 9 station objective structured clinical examination.	The Patient Partner in Arthritis Program™ trained patient educators in a musculoskeletal examination course.	Musculoskeletal examination skills.	10	5 patient educators and 5 silent patients; patients with arthritis		Active teacher	62
**Towle, A. et al.**	2014	The Clinical Teacher	The expert patient as teacher: an interprofessional Health Mentors programme.	Canada	Not specified	Case study	Description of curriculum development	Interprofessional Education (IPE), Health Mentors Programme	Report on evaluation of ongoing programme		Patients with a wide range of conditions, including HIV/AIDS, arthritis, multiple sclerosis, spinal cord injury, cerebral palsy and mental health problems	Community organizations facilitate mentor recruitment	Active teacher, mentorship/coach	290
**Oswald, A.E. e.al.**	2011 a	BMC Medical Education	The impact of trained patient educators on musculoskeletal clinical skills attainment in pre-clerkship medical students	Canada	Are there differences in physical examination skills between physician and patient taught students?	Randomized controlled clinical trial	Objective Structured Clinical Examination and questionnaires.	The Patient Partner in Arthritis Program™ trained patient educators in a musculoskeletal examination course.	Musculoskeletal examination skills.		Volunteer patient educators with arthritis		Active teacher	83

For peer review only

**Jaworsky, D. et al.**	2017	AIDS Care	The role of people living with HIV as patient instructors – reducing stigma and improving interest around HIV care among medical students	Canada	What is the impact of patient-facilitated education on reducing HIV-related stigma?	Before and after study	Health Care Provider HIV/AIDS Stigma Scale (HPASS) questionnaire	Simulated clinical encounter in which medical students provided HIV pre- and post-test counselling.	Stigma on HIV and comfort in providing HIV care	16	People living with HIV/AIDS	PHAs were recruited through a community partner, the Toronto People With AIDS Foundation	Active teacher	67
**Haq, I. Fuller, J., Dacre, J.**	2006	Rheumatology	The use of patient partners with back pain to teach undergraduate medical students	UK	What is the impact of teaching about back pain to medical students using trained patient partners?	Randomized controlled clinical trial	Objective Structured Clinical Examination and questionnaires.	Teaching about back pain to medical students by trained patient partners, during at 5-week rheumatology/orthopaedics firm.	Musculoskeletal examination skills.	4	Three male patients and one female , age 29–56. Ankylosing spondylitis and chronic back pain with intermittent sciatica.		Active teacher	60
**Collins, L. et al.**	2011	Journal of Interprofessional Care	Transforming chronic illness care education: A longitudinal interprofessional mentorship curriculum	USA	What are student reflections on the impact of a longitudinal mentor program with a chronic condition?	Qualitative description	Students reflection assays.	Interprofessional Health Mentors Program and Chronic Care Model	Student perspectives on the programme and chronic illness		Patients with chronic illness or disability		Coach/mentorship	60
**Raj, N. et al.**	2006	Rheumatology	Undergraduate musculoskeletal examination teaching by trained patient educators—a comparison with doctor-led teaching	UK	How do hand and knee examination skills gained by undergraduates taught either by trained patient educators and physicians compare?	Randomized controlled clinical trial	Objective Structured Clinical Examination and questionnaires.	Students were taught an established core skills set in small group workshops. Students	Musculoskeletal examination skills.	12	11 female, 1 male, age 34–79. Rheumatoid arthritis, osteoarthritis, inflammatory arthritis.		Active teacher	50
**Tierney, W.M.**	2018	Journal of General Internal Medicine	Use of Stakeholder Focus Groups to Define the Mission and Scope of a new Department of Population Health.	USA	Not specified	Case study	Description of curriculum development	Input for strategic development of department of population health in new medical school	Consensus on focus areas for the school's curriculum	13	Local community non-profit organizations	People recruited from local community organizations	Curriculum development	
**Duggan, A. et al.**	2009	Journal of Health Communication	What can I learn from this interaction? A qualitative analysis of medical student self-reflection and learning in a standardized patient exercise about disability	USA	How does reflective practice about patients with disability promote relationship-centered care?	Qualitative - Grounded theory design	Videotaped feedback sessions between student en simulated patient with disability	Simulated patient encounter with a patient with an apparent disability.	Student reflections and insights gained	12	Patients with an apperant disability; included Multiple Sclerosis, Juvinile rheumathoid arthritis, traumatic spinal cord injury, neurodegenerative disorders, cerebral palsy, muscular dystrophy, blindness, paraplegia, quadriparesis, respiratory failure, NF and mobility constraint		Standardized Patient Educator	138
**Jha, V. et al.**	2009	Medical Education	What educators and students really think about using patients as teachers in medical education: a qualitative study	UK	What are the views and experiences of medical tutors and students of involving patients?	Qualitative description	Semi-structured interviews	Medical school - general	Student and medical tutor views and experiences on patients as teachers in medical training				Curriculum development, active teacher, assessor	28
**Alahlafi, A., Burge, S.**	2005	British Medical Journal	What should undergraduate medical students know about psoriasis? Involving patients in curriculum development: modified Delphi technique	UK	Not specified	Case study	Literature review and modified Delphi technique.	Medical school - general	Consensus among patients/physicians/nurses on content of the psoriasis workshop.	20	Patients with psoriasis		Curriculum development	
**Stagg, P., Rosenthal, D.R.**	2012	Rural and Remote Health	Why community members want to participate in the selection of students into medical school	Australia	What motivates community members to participate in the selection of medical students?	Qualitative description	Semi-structured interviews	Community member participation in selection of medical students	Patient experience and perceptions	8	Community Liaison Committee members		Student selection	

For peer review only

PRISMA 2009 Checklist

Section/topic	#	Checklist item	Reported on page #
**TITLE**			
Title	1	Identify the report as a systematic review, meta-analysis, or both.	1
**ABSTRACT**			
Structured summary	2	Provide a structured summary including, as applicable: background; objectives; data sources; study eligibility criteria, participants, and interventions; study appraisal and synthesis methods; results; limitations; conclusions and implications of key findings; systematic review registration number.	2
**INTRODUCTION**			
Rationale	3	Describe the rationale for the review in the context of what is already known.	3
Objectives	4	Provide an explicit statement of questions being addressed with reference to participants, interventions, comparisons, outcomes, and study design (PICOS).	3
**METHODS**			
Protocol and registration	5	Indicate if a review protocol exists, if and where it can be accessed (e.g., Web address), and, if available, provide registration information including registration number.	3
Eligibility criteria	6	Specify study characteristics (e.g., PICOS, length of follow-up) and report characteristics (e.g., years considered, language, publication status) used as criteria for eligibility, giving rationale.	3
Information sources	7	Describe all information sources (e.g., databases with dates of coverage, contact with study authors to identify additional studies) in the search and date last searched.	3
Search	8	Present full electronic search strategy for at least one database, including any limits used, such that it could be repeated.	3, Supplementary
Study selection	9	State the process for selecting studies (i.e., screening, eligibility, included in systematic review, and, if applicable, included in the meta-analysis).	3
Data collection process	10	Describe method of data extraction from reports (e.g., piloted forms, independently, in duplicate) and any processes for obtaining and confirming data from investigators.	3
Data items	11	List and define all variables for which data were sought (e.g., PICOS, funding sources) and any assumptions and simplifications made.	-
Risk of bias in individual studies	12	Describe methods used for assessing risk of bias of individual studies (including specification of whether this was done at the study or outcome level), and how this information is to be used in any data synthesis.	-
Summary measures	13	State the principal summary measures (e.g., risk ratio, difference in means).	-
Synthesis of results	14	Describe the methods of handling data and combining results of studies, if done, including measures of consistency (e.g., I^2) for each meta-analysis.	3

For peer review only

For peer review only - http://bmjopen.bmj.com/site/about/guidelines.xhtml

PRISMA 2009 Checklist

Section/topic	#	Checklist item	Reported on page #
Risk of bias across studies	15	Specify any assessment of risk of bias that may affect the cumulative evidence (e.g., publication bias, selective reporting within studies).	-
Additional analyses	16	Describe methods of additional analyses (e.g., sensitivity or subgroup analyses, meta-regression), if done, indicating which were pre-specified.	-
**RESULTS**			
Study selection	17	Give numbers of studies screened, assessed for eligibility, and included in the review, with reasons for exclusions at each stage, ideally with a flow diagram.	3,4
Study characteristics	18	For each study, present characteristics for which data were extracted (e.g., study size, PICOS, follow-up period) and provide the citations.	Supplementary
Risk of bias within studies	19	Present data on risk of bias of each study and, if available, any outcome level assessment (see item 12).	-
Results of individual studies	20	For all outcomes considered (benefits or harms), present, for each study: (a) simple summary data for each intervention group (b) effect estimates and confidence intervals, ideally with a forest plot.	-
Synthesis of results	21	Present results of each meta-analysis done, including confidence intervals and measures of consistency.	-
Risk of bias across studies	22	Present results of any assessment of risk of bias across studies (see Item 15).	-
Additional analysis	23	Give results of additional analyses, if done (e.g., sensitivity or subgroup analyses, meta-regression [see Item 16]).	-
**DISCUSSION**			
Summary of evidence	24	Summarize the main findings including the strength of evidence for each main outcome; consider their relevance to key groups (e.g., healthcare providers, users, and policy makers).	9
Limitations	25	Discuss limitations at study and outcome level (e.g., risk of bias), and at review-level (e.g., incomplete retrieval of identified research, reporting bias).	10
Conclusions	26	Provide a general interpretation of the results in the context of other evidence, and implications for future research.	10
**FUNDING**			
Funding	27	Describe sources of funding for the systematic review and other support (e.g., supply of data); role of funders for the systematic review.	10

From: Moher D, Liberati A, Tetzlaff J, Altman DG, The PRISMA Group (2009). Preferred Reporting Items for Systematic Reviews and Meta-Analyses: The PRISMA Statement. PLoS Med 6(7): e1000097. doi:10.1371/journal.pmed1000097

For more information, visit: **www.prisma-statement.org**.

For peer review only - http://bmjopen.bmj.com/site/about/guidelines.xhtml
